# Whole genome and whole transcriptome genomic profiling of a metastatic eccrine porocarcinoma

**DOI:** 10.1038/s41698-018-0050-5

**Published:** 2018-03-19

**Authors:** My Linh Thibodeau, Melika Bonakdar, Eric Zhao, Karen L. Mungall, Caralyn Reisle, Wei Zhang, Morgan H. Bye, Nina Thiessen, Dustin Bleile, Andrew J. Mungall, Yussanne P. Ma, Martin R. Jones, Daniel J. Renouf, Howard J. Lim, Stephen Yip, Tony Ng, Cheryl Ho, Janessa Laskin, Marco A. Marra, Kasmintan A. Schrader, Steven J. M. Jones

**Affiliations:** 1 Department of Medical Genetics, University of British Columbia, C201–4500 Oak Street, Vancouver, BC V6H 3N1 Canada; 2 Canada’s Michael Smith Genome Sciences Centre, British Columbia Cancer Agency, 100–570 West 7th Avenue, Vancouver, BC V5Z 4S6 Canada; 3 Department of Medical Oncology, British Columbia Cancer Agency, 600 West 10th Avenue, Vancouver, BC V5Z 4E6 Canada; 4 Department of Pathology & Laboratory Medicine, Vancouver General Hospital, 910 West 10th Avenue, Vancouver, BC V5Z 1M9 Canada; 5 Hereditary Cancer Program, Department of Medical Genetics, British Columbia Cancer Agency, 614–750 West Broadway, Vancouver, BC V5Z 1H5 Canada

## Abstract

Eccrine porocarcinomas (EPs) are rare malignant tumours of the intraepidermic sweat gland duct and most often arise from benign eccrine poromas. Some recurrent somatic genomic events have been identified in these malignancies, but very little is known about the complexity of their molecular pathophysiology. We describe the whole genome and whole transcriptome genomic profiling of a metastatic EP in a 66-year-old male patient with a previous history of localized porocarcinoma of the scalp. Whole genome and whole transcriptome genomic profiling was performed on the metastatic EP. Whole genome sequencing was performed on blood-derived DNA in order to allow a comparison between germline and somatic events. We found somatic copy losses of several tumour suppressor genes including *APC*, *PTEN* and *CDKN2A*, *CDKN2B* and *CDKN1A*. We identified a somatic hemizygous *CDKN2A* pathogenic splice site variant. De novo transcriptome assembly revealed abnormal splicing of *CDKN2A* p14^ARF^ and p16^INK4a^. Elevated expression of oncogenes *EGFR* and *NOTCH1* was noted and no somatic mutations were found in these genes. Wnt pathway somatic alterations were also observed. In conclusion, our results suggest that the molecular pathophysiology of malignant EP features high complexity and subtle interactions of multiple key genes. Cell cycle dysregulation and *CDKN2A* loss of function was found to be a new potential driver in EP tumourigenesis. Moreover, the combination of somatic copy number variants and abnormal gene expression perhaps partly related to epigenetic mechanisms, all likely contribute to the development of this rare malignancy in our patient.

## Introduction

Eccrine porocarcinomas (EPs) are very rare malignant tumours of the intraepidermic sweat gland duct.^1^ In the United States, the age-adjusted incidence rate ratio of porocarcinoma is 0.4 case per 1 million person-year and median age at diagnosis is 75 years.^2^ Very little is known about the molecular pathophysiology of this tumour and only targeted tumour sequencing of EPs has been published to date. Harms et al.^3^ recently suggested that porocarcinomas feature recurrent somatic *HRAS* and *EGFR* gain of function (GoF) mutations and various tumour suppressor genes loss of function (LoF) mutations. A *PIK3CA* somatic GoF mutation has been reported in one case of porocarcinoma.^4^ We describe the whole genome and whole transcriptome profiling of metastatic EP in a 66-year-old male with a previous history of localized EP of the scalp.

## Clinical description

**Fig. 1:**
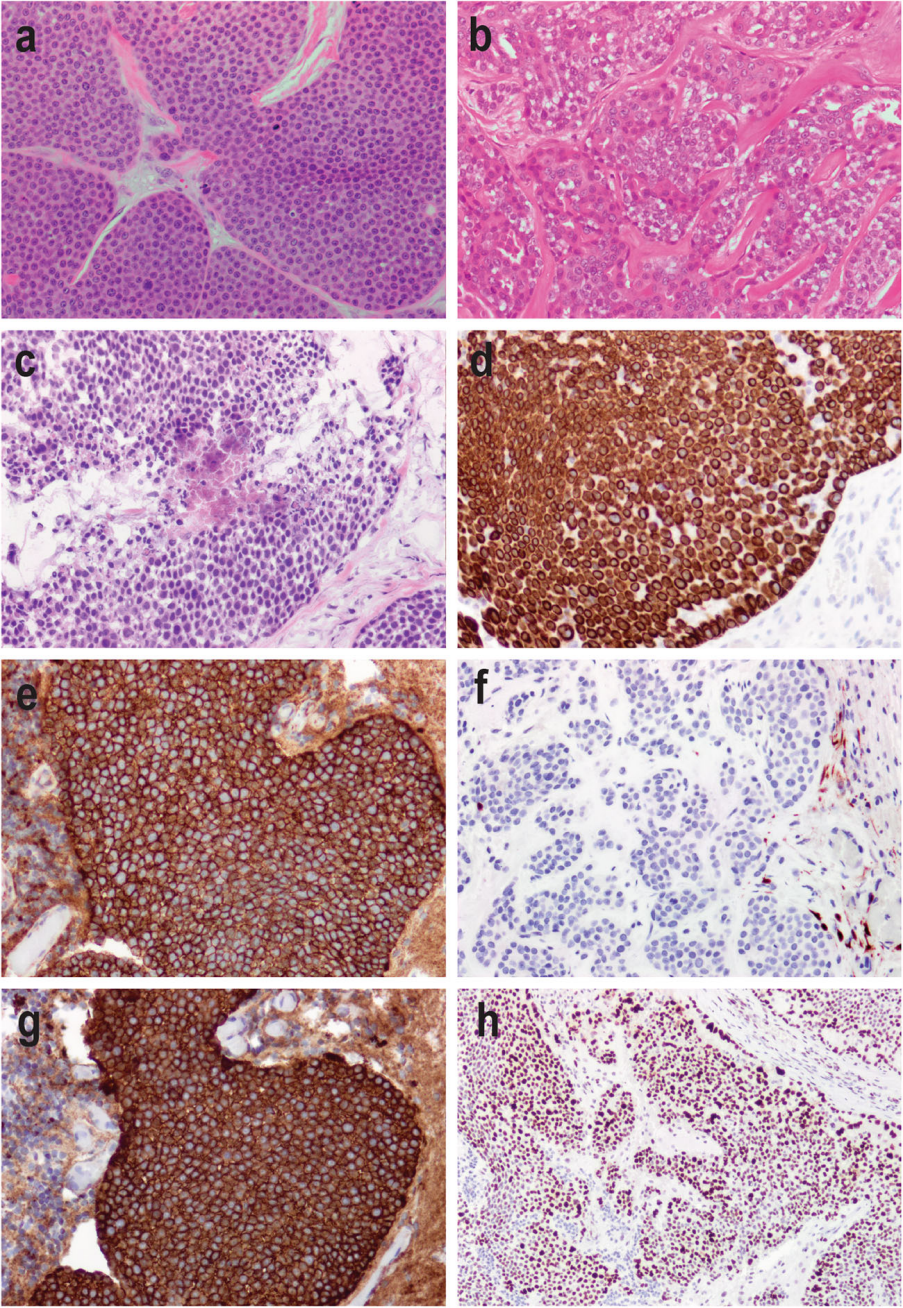
Histology and immunochemistry profile of poroid neoplasm. **a** Hematoxylin & eosin (H&E) stained section of primary scalp lesion. **b** H&E-stained section of subsequent metastatic left neck lesion. **c** H&E-stained section of subsequent metastatic cerebellar tumour. Immunohistochemistry of the cerebellar tumour for **d** CK5, **e** β-catenin, **f** p16, **g** EGFR, and **h** p53. All images are shown at 200× magnification

A male patient presented at the age of 64 years with a bleeding left-sided scalp lesion. The lesion was resected with clear margins and pathology examination concluded to a diagnosis of EP. Eighteen months later, the patient presented with left cervical lymphadenopathies and the incisional biopsy revealed pathology features in keeping with EP recurrence. Oncological management included left neck radical dissection with en bloc excision of the left occipital EP mass, followed by local radiation therapy. Post-treatment fluorodeoxyglucose-positron emission tomography (PET) scan imaging was negative for evidence of distant metastasis. Nine months later, the follow-up PET scan showed a tracer avid left supraclavicular node and pathology examination confirmed metastatic EP. Systemic therapies were considered, but not pursued, as the site of recurrence was resected and there was a lack of scientific literature supporting efficacy of such therapies in EP management. Recognizing that there was no potential quality of life benefit, the patient opted for surveillance. Shortly after, the patient acutely developed cerebellar signs and his brain magnetic resonance imaging with gadolinium showed an enhancing right cerebellar lesion measuring 1.9 × 3.3 × 2.1 cm^3^. Right occipital craniotomy and cerebellar metastasis surgical resection was performed for symptomatic relief. Pathology of the resected tissue confirmed metastatic EP (Fig. 1). Two months after, the patient passed away from progressive central nervous system involvement with leptomeningeal spread. For a more detailed clinical description, please refer to Supplementary Information.

## Results

### Pathology

Pathological examination of the primary scalp lesion showed a monotonous proliferation with epidermal attachment and elevated mitotic activity, consistent with a poroid neoplasm (Fig. 1a). Examination of the subsequent left neck resection specimen showed very similar histologic features, with metastatic tumour cells in 38 of 49 neck lymph nodes examined, confirming the diagnosis of EP (Fig. 1b). The subsequent cerebellar metastatic tumour showed identical histological features as the primary lesion and the neck resection specimen, consisting of a highly infiltrative carcinoma composed of epithelioid cells with eosinophilic and focally clear cytoplasm, arranged in sheets and nodules, within the cerebellar parenchyma, including wide spread central necrosis and areas of discohesive growth (Fig. 1c). CK5 immunohistochemistry showed diffuse cytoplasmic staining in the tumour cells, consistent with the expected immunoprofile for EP (Fig. 1d). β-catenin staining showed strong membranous and cytoplasmic positivity with no nuclear staining, supporting the absence of canonical Wnt pathway activation (Fig. 1e). Staining for p16, the protein product of *CDKN2A* (p16^INK4a^), was absent in the tumour (Fig. 1f). EGFR immunostaining showed diffuse membranous staining (Fig. 1g), while staining for p53 showed variable nuclear positivity, consistent either with wild-type or missense *TP53* status (Fig. 1h).

### Somatic profiling

Tumour genomic profiling was performed on a lymph node metastasis from the wide left neck biopsy (2015) at the site where previous left radical neck dissection with en bloc excision of left occipital porocarcinoma was performed (2013). Unless otherwise specified, the tumour expression percentile comparison was against The Cancer Genome Atlas (TCGA) average of all cancers (disease comparator).^5^ Somatic profiling included assessment of protein coding mutations (small mutations and structural rearrangements) and mutational burden, copy number analysis, gene expression analysis from transcriptome data and mutational signatures (please refer to Methods and Supplementary Information for more details). There were 38 protein coding somatic small mutations, including 35 (44th percentile) non-synonymous single-nucleotide variants (SNVs) and 3 indels (38th percentile) and there were 40 (21st percentile among our local database of 584 diverse cancer cases) structural variants (SVs). Two expressed SV fusions due to large deletion events were identified: *RNF13-PAK2* and *PIK3R1*-*YTHDC2* (see section below on copy number and structural variants). There were regional copy gains (chromosome 3) and losses (chromosomes 1, 3, and 5), as well as chromosome-wide copy loss of chromosome 6 (Supplementary Figs. S1 and S2). There were no large regions of loss of heterozygosity (LOH). The transcriptome Spearman's correlation showed the highest correlation with squamous cancers, specifically oesophageal squamous carcinoma (Supplementary Fig. S3). The somatic SNV profile revealed a best-fit mutation signature model that comprised of signatures 1, 8, 9, and 16 (Supplementary Figs. S4, S5 and S6).^6^ Signature 1 is associated with age, and is ubiquitous across cancer types. Refer to Supplementary Information and Supplementary Results (Tables S1, S2, S3 and S4) for more details.

### Cell cycle regulation

*CDK6* had elevated expression (96th percentile). The tumour had deletion copy loss of one *CDKN2A* allele and a somatic splice site acceptor mutation located at the last base from intron 1, at its junction with exon 2 (chr9:21971208C>T, GRCh37; c.151−1G>A, NM_000077.4; c.194−1G>A, NM_058195.3; COSM127095) on the remaining allele (http://cancer.sanger.ac.uk).^7^ The variant was absent in germline (0/30 reads) and hemizygous in the tumour (19/19 reads) (Supplementary Table S2). Alterations of this splice site are present in ClinVar (rs730881677, c.151-1G>T and c.151-1G>C, NM_000077.4) and associated with familial melanoma.^8^

**Fig. 2:**
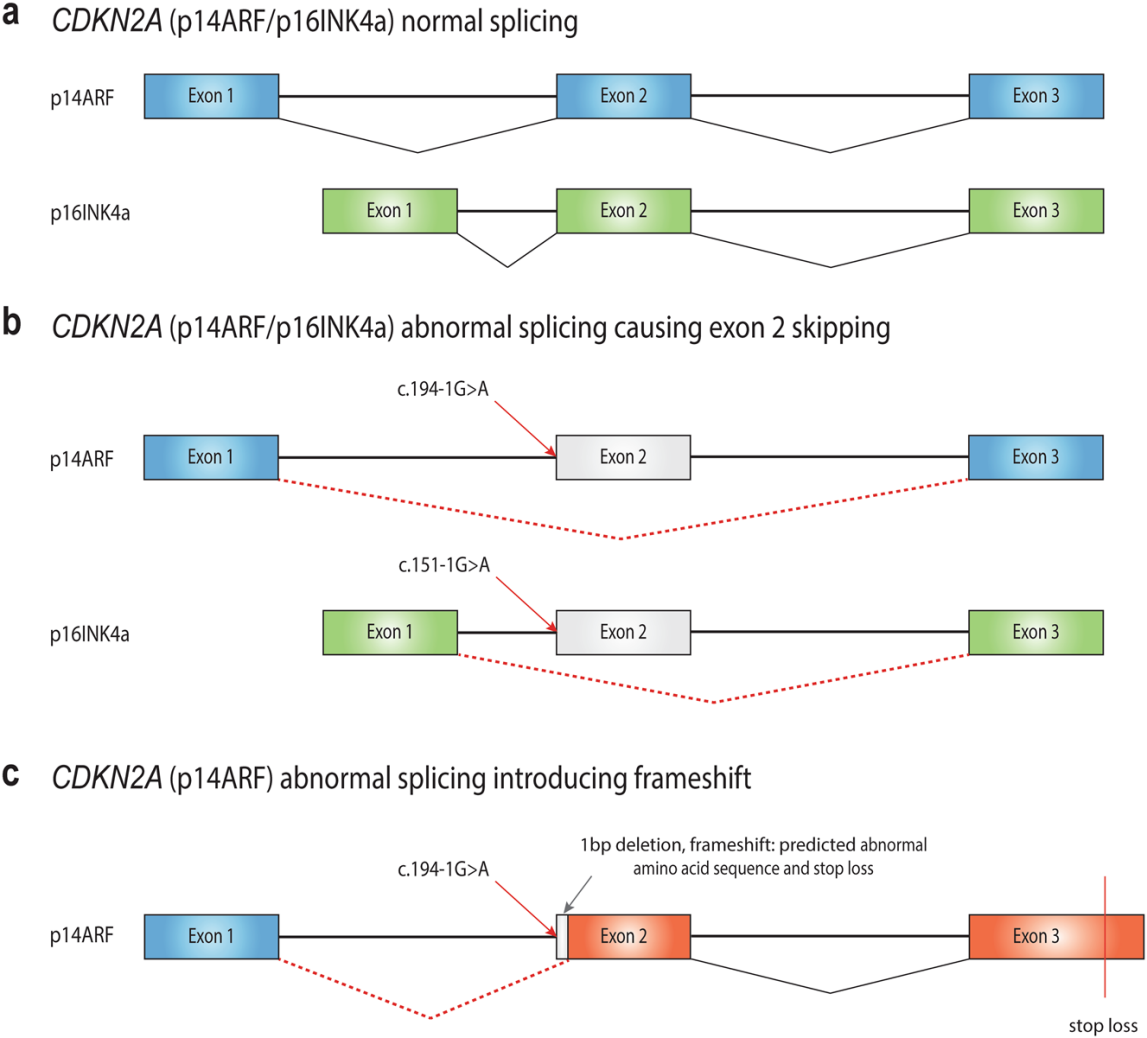
*CDKN2A* splicing. **a** *CDKN2A* (p14^ARF^/p16^INK4a^) normal splicing. **b** *CDKN2A* exon 2 skipping caused by the somatic splice site mutation (p14^ARF^c.194-1G>A, NM_05895.3; p16^INK4a^ c.151-1G>A, NM_000077.4). **c** *CDKN2A* (p14) abnormal splicing caused by the c.194−1G>A (NM_058195.3) somatic mutation leading to 1 base pair deletion and a frameshift

De novo transcriptome assembly revealed that the *CDKN2A* splice site variant causes exon 2 skipping of both the p14^ARF^ transcript (ENST00000361570; NM_058195.3; supported by 109 reads) and the p16^INK4a^ (ENST00000304494; NM_000077.4; supported by 21 reads) transcripts (Fig. 2). A rarer second splicing abnormality was identified in a minority of p14^ARF^ transcripts (four reads) and leads to the removal of the first base of exon 2 (chr9:21971207, hg19), resulting in a frameshift (Fig. 2c).

The exon-specific collapsed transcript expression (cumulative coverage across each exon) showed relatively low levels of expression in reads per kilobase per million mapped reads at the corresponding genomic area of exon 2 for both ENST00000361570 (p14^ARF^) and ENST00000304494 (p16^INK4a^) transcripts (Supplementary Table S2 and Figs. S7 and S8), consistent with exon 2 skipping.

### Cell growth, cell survival and Wnt pathway

Tumour suppressor copy loss of *PTEN* was observed. When compared to TCGA average (Supplementary Information for details), we noted elevated expression for *KRAS*, *EGFR* and *NOTCH1* (Table 1). Amplification of *GSK3B* (glycogen synthase kinase 3β) was seen (four copies in total), associated with high expression compared to TCGA average (100th percentile, Table 1). *WNT10A* was highly expressed (Table 1), but no genomic causal event was identified. Copy losses of *APC*, *CTNNB1*, *WNT5A* and *WNT2B* were identified, but their transcriptome expression was average.

**Table 1:** RNA expression metrics of selected genes (diploid model)

Gene	Copy number change (diploid model)	All TCGA		ESCA_SCC TCGA		All TCGA (matched normal)		Bodymap
		%ile	kIQR	%ile	kIQR	%ile	kIQR	Mean FC
*APC*	−1 (DLOH)	84	1.1	70	0.41	86	0.88	−1.17
*BAP1*	−1 (DLOH)	1	−1.5	0	−1.77	0	−2.33	−1.79
*BRAF*	0	94	1.7	79	0.73	100	2.06	1.25
*CDK4*	0	2	−0.96	4	−1.1	13	−0.91	1.48
*CDK6*	0	96	4.25	89	1.08	100	9.78	7.59
*CDKN1A*	−1 (DLOH)	60	0.2	56	0.06	56	0.11	1.44
*CDKN2A*	−1 (DLOH)	60	0.15	72	0.56	99	4.35	4.67
*CDKN2B*	−1 (DLOH)	96	3.74	90	1.43	91	1.78	3.91
*CTNNB1*	−1 (DLOH)	86	1.06	97	2.49	91	1.5	1.73
*CYLD*	0	65	0.28	56	0.1	53	0.04	−1.59
*DPH3*	0	95	1.91	100	3.36	95	1.3	−1.01
*E2F1*	0	31	−0.3	9	−0.73	92	1.5	1.98
*E2F2*	0	46	−0.08	6	−0.99	82	2.24	1.45
*E2F3*	−1 (DLOH)	48	−0.03	47	−0.05	91	1.13	1.09
*EGFR*	0	99	17.91	93	3.62	100	29.5	16.72
*ERBB2*	0	22	−0.47	3	−0.68	10	−0.79	1.77
*EZH2*	0	42	−0.14	14	−0.63	90	2.35	1.57
*FZD1*	0	90	2.28	92	2.15	92	1.97	3.21
*FZD6*	0	97	2.78	53	0.04	100	3.95	5.25
*FZD7*	0	98	5.58	93	1.52	97	4.43	4.18
*GSK3B*	+2 (ASCNA)	100	5.08	97	2.22	100	8.58	3.62
*HRAS*	0	7	−0.69	0	−1.09	14	−0.7	1.05
*JAG1*	0	99	5.62	83	1.02	100	8.97	6.42
*KRAS*	0	96	2.45	90	1.24	100	4.41	1.83
*MDM2*	0	97	3.31	97	4.19	99	5.89	2.86
*MDM4*	0	93	2.08	90	2.07	100	3.26	−1.07
*MUTYH*	0	1	−0.97	2	−1.37	5	−0.91	−1.14
*NOTCH1*	0	96	3.16	82	0.96	100	4.81	5.35
*PIK3CB*	+2 (ASCNA)	89	1.19	82	0.98	87	1.05	1.76
*PTEN*	−1 (DLOH)	80	0.69	76	0.64	66	0.33	1.14
*RB1*	0	95	1.82	64	0.29	100	3.31	2.26
*TP53*	0	64	0.29	56	0.16	89	1.19	1.75
*TGFB1*	0	79	0.73	18	−0.49	94	1.75	1.77
*TGFBR1*	0	94	1.79	90	1.45	97	2.05	1.86
*TGFBR2*	0	99	3.84	100	6.67	81	0.82	1.15
*WNT10A*	0	96	8.33	89	1.54	100	14.41	18.52
*WNT5A*	−1 (DLOH)	83	1.09	33	−0.13	91	2.12	2.9

*TCGA* The Cancer Genome Atlas, *ASCNA* allele-specific copy number alteration, *DLOH* deletion loss of heterozygosity, *ESCA_SCC* oesophageal squamous cell carcinoma, *FC* fold change, *kIQR* number of inter-quartile range intervals away from the median, *%ile* percentile

### Copy number variants and structural variants

The copy number variants (CNVs) of note were copy losses of *APC*, *CDKN2A/B*, *PTEN*, *CTNNB1*, *FOXP1*, *MITF* and *BAP1* and copy gains of *GSK3B*, *PIK3CB* and *ATR* (refer to Supplementary Table S1B and Figs. S1 and S2 for CNV details). Two SVs are expressed in the transcriptome. A 46 Mb deletion on chromosome 3 (chr3:149653091–196530353, hg19) leads to the fusion of *RNF13* and *PAK2* (Supplementary Fig. S9). *PAK2* has a two-copy gain and is highly expressed (99th percentile). A 45 Mb deletion on chromosome 5 (chr5:67564688–112859542, hg19) leads to the fusion of *PIK3R1* and *YTHDC2* (Supplementary Fig. S10) and *PIK3R1* has high expression (87th percentile). See Supplementary Information, Tables S1B and S1D and Figs. S9 and S10 for details on genomic events and potential biological implications.

## Discussion

Whole genome and whole transcriptome analysis of this case of metastatic EP provided insight into the complex molecular pathophysiology of this rare tumour. Overall, somatic SNV, CNV/LOH and SV were scarce when compared to other cutaneous tumours or even non-cancerous sun-exposed skin.^9^ However, some key components of cell cycle regulation and Wnt pathways were somatically altered, and will be further discussed below.

Previously reported cases of EP have been characterized by LOH, *TP53* alterations and a paucity of cytogenetically detectable abnormalities when compared to other cutaneous squamous cell carcinomas.^10^ This contrasts the findings in our case, where multiple disruptions to *CDKN2A* (loss of one copy and somatic hemizygous splice site acceptor mutation) were identified, but no *TP53* mutation.

While the global gene expression level of *CDKN2A* was not obviously perturbed (Table 1), p16^INK4a^ isoform had decreased expression (Supplementary Table S2) and *CDNK2A* splice site variant causes abnormally spliced p14^ARF^and p16^INK4a^ transcripts (Fig. 2, Supplementary Table S2 and Fig. S8). Moreover, p16^INK4a^ protein (*CDKN2A*) immunohistochemical (IHC) staining was absent in our case, in keeping with absence of a functional protein product (Fig. 1f). Interestingly, Tsujita et al.^11^ found p16^INK4a^ staining to be moderate to strongly positive in 16/17 eccrine poroma and focally and diffusely positive in 4/4 porocarcinoma tumour samples. Germline *CDKN2A* mutations are associated with significant predisposition to pancreatic cancer and hereditary melanoma.^12^ *CDKN2A* somatic mutations are seen not only in melanoma and non-melanoma skin cancers,^13^ but also in tumours arising from the central nervous system, the pleura and the oesophagus.^7^ Combined with somatic *TP53* alterations, *CDKN2A* LoF is a frequent feature of cutaneous squamous cell^14^ and oesophageal carcinomas.^15^ Intragenic, epigenetic and copy loss mutations of *CDKN2A* and *CDKN2B* play an important role in oesophageal squamous cell oncogenesis.^16^ Our porocarcinoma transcriptome profiling displays the highest correlation with TCGA oesophageal squamous carcinoma expression data, which may be explained by the epithelial origin of both cancer types and the genomic events described above leading to cell cycle dysregulation and oncogenesis (Supplementary Table S1 and Figs. S2, S3, S7 and S8).

Whole genome porocarcinoma sequencing revealed a somatic focal copy loss at 3p21.3, which encompasses *BAP1*, another cell cycle gene frequently mutated in inherited and sporadic melanomas.^17^ Together, these findings indicate that cell cycle dysregulation likely plays a role in our patient’s EP oncogenesis.

*PTEN* copy loss was observed in our case. Somatic *PTEN* copy loss and mutations have not been reported in human porocarcinoma, but are relatively frequent events in melanoma^18^ and squamous cell carcinomas.^19^ A mouse model of squamous cell carcinoma of the skin showed that epidermal *Pten* knockout leads to skin tumour formation via increased autocrine fibroblast growth factor signalling.^20^ Suzuki et al.^21^ demonstrated that combining in vitro and animal models provides critical insight on skin tumour development. Suzuki et al.^21^ created a keratinocyte-specific *Pten* Cre-loxP knockout mouse model. All k5Pten^flox/flox^ mice and 23% of k5Pten^flox/+^ mice developed squamous papillomas and squamous cell carcinomas, but one mouse developed an eccrine sweat gland adenocarcinoma (or EP), suggesting that PTEN loss may be a critical and early event in the development of EP.^21^ Our EP tumour displays a single deletion copy loss of *PTEN*, but *PTEN* expression was unremarkable (80th percentile for all TCGA cancers).

We observe several alterations of the PI3K-AKT-RAS pathway at the genomic and transcriptome levels. Somatic copy loss of *PTEN*, a regulator of PI3K-AKT-RAS pathway, may contribute to *EGFR* overexpression. Although the expression profile of AKT genes is unremarkable, this might be explained by the effects of downstream *GSK3B* copy gains (four copies in total) and *GSK3B* high expression compared to TCGA average (100th percentile, Table 1).

*EGFR*, which has been hypothesized to be an oncogenic driver in porocarcinoma,^3^ has elevated expression (99th percentile for all TCGA cancers, 93rd percentile for TCGA-ESCA and 16.72-fold change from the mean Illumina BodyMap), but we identified no mutation in *EGFR* and EGFR ligands expression was unremarkable (Supplementary Information and Table S1b).^22,23^ We found one deletion copy loss of *LRIG1*, a known *EGFR* inhibitor, in our EP tumour.^24^ EP EGFR IHC showed strong positivity (Fig. 1g), also supporting the overrepresentation of *EGFR* at the functional cellular level. Fluorescent in situ hybridization (FISH) and immunochemistry studies suggest that EGFR inhibitors could hypothetically inhibit growth of metastatic adnexal tumours.^4^ Although such therapeutic agents have not been studied clinically, they represent promising treatment avenues deserving further studies. *SOS1/SOS2* overexpression combined with increased *EGFR* activity most likely explain the RAS pathway activation with high expression of *KRAS* and *BRAF*. As EGFR IHC has many caveats and no *EGFR* genomic alterations were detected, combining the transcriptome data and pathway analysis allowed us to identify EGFR as a potential therapeutic target to consider in EP management. EGFR phosphorylation assays may aid in determining if *EGFR* overexpression alone can be used as a marker for response to EGFR inhibitors or if EGFR site-specific phosphorylation may be associated with EGFR inhibitor response in a subset of wild-type *EGFR* cases, such as demonstrated in non-small-cell lung cancer.^25^ EP *KRAS* overexpression was observed (Table 1) and is of interest given the previously described oncogenic role of RAS family genes in EP.^3^ *NOTCH1*, which can contribute to RAS pathway over-activation, was also overexpressed (Table 1). Recently, *KRAS* and *PIK3CB* signalling have been noted to have a direct relationship in oral squamous cell carcinomas and these oncogenes may become therapeutic targets in the future.^26^ Overall, unlike several well-characterized tumour types, such as oesophageal carcinomas or skin melanomas, the genomic profiling of our EP tumour is unique and does not fit any specific pattern. In our case, the patient’s clinical situation deteriorated rapidly after resection of his cerebellar metastases due to leptomeningeal spread and systemic therapy was not feasible, but advances in understanding EP pathophysiology coupled with novel targeted agents may soon offer better therapeutic strategies to treat patients with this rare tumour.

## Conclusions

Our results suggest that the molecular pathophysiology of malignant EP features high complexity and subtle interactions of multiple key genes. Cell cycle dysregulation and *CDKN2A* LoF was found to be a new potential driver in EP tumourigenesis. Moreover, the combination of somatic copy number variants and abnormal gene expression, perhaps partly related to epigenetic mechanisms, all likely contributes to the development of this rare malignancy in our patient.

Given *GSK3B* amplification and high expression and copy losses of *APC* and *CTNNB1*, our data raise the possibility of Wnt pathway contribution to EP pathogenesis. No clear “targetable” pathway or genomic alteration was identified in our EP tumour, but given the rarity of this tumour as well as paucity of EP genomic data available, determining the utility of genomic profiling in guiding EP management requires additional comprehensive genomic studies. Specifically, further research is necessary to appreciate if EP tumours display recurrent and potentially targetable mutations, or if such tumours are molecularly heterogeneous and difficult to characterize. Moreover, complementary functional studies such as proteomics and detailed immunochemistry are needed to improve genomic profiling interpretation and assist in delineating the molecular pathophysiology of EP.

## Methods

### Clinical sample

This research project was approved by the University of British Columbia Cancer Agency (BCCA) Research Ethics Board (REB) (protocol H14-00681). Informed written consent was obtained from the patient for tumour profiling using RNA-seq (tumour) as well as whole genome sequencing (tumour and blood). The use of datasets is allowed for research reports and scientific publications. The protocol allows for data to be not only used for research reports and scientific publication, but also to be made available to named investigators of institutions who agree by a data transfer agreement stating they will honour the same ethical and privacy principles required by the BCCA REB. Following informed consent, the patient underwent imaging-guided left neck lymph nodes metastases biopsies as part of the Personalized OncoGenomics trial at the British Columbia Cancer Agency (Clinicaltrials.gov ID:NCT02155621). Peripheral venous blood samples were also obtained and leucocytes were isolated for germline DNA analysis. DNA and RNA extraction, library construction and sequencing were performed according to previously described standard protocols (Supplementary Information— Methods).^27,28^ Methods were conducted in accordance to the review board-approved protocols.

### Sequencing and bioinformatics

Paired-end reads were generated on an Illumina HiSeq2500 sequencer (Illumina Inc., San Diego, CA, USA; http://www.illumina.com/). Tumour biopsy and peripheral blood samples were sequenced to a depth of 90× and 42×, respectively, using established PCR-free whole genome sequencing and ssRNA-seq protocols previously described.^27,28^ Reads were aligned to the hg19 (GRCh37) reference using BWA-MEM v0.7.6.^29^ For both germline and somatic genomic variants, mpileup and varFilter from SAMtools (v0.1.17) were used for variant calling and filtering.^30,31^ Tumour content and ploidy model estimations were derived from sequencing data through analysis of the CNA (copy number alteration) ratios and allelic frequencies of individual chromosomes; the best fit for this tumour was a diploid model at 66% tumour content.^32,33^ Regions of CNV and LOH were identified using Hidden Markov model-based approaches CNAseq (v0.0.8)^32^ and APOLLOH (v0.1.2),^34^ respectively. Somatic variants (SNV, indels, structural variants) were called on genomic and allele counts (reference vs. alternate) from DNA and RNA-seq tumour data (compared against blood genomic data) using a combination of tools: SAMtools (v0.1.17),^30^ MutationSeq (v4.3.5)^35^ and Strelka (v1.0.6)^36^ for SNVs; Strelka (v1.0.6)^36^ and Trans-ABySS (v1.4.10)^37,38^ for indels; de novo assembly and annotation of genomic and transcriptomic data with ABySS (v1.3.4),^39^ Trans-ABySS (v1.4.10),^37,38^ deFuse (v0.6.2)^40^ and MAVIS (manuscript in preparation) for structural variants and fusion genes. Somatic mutations were classified by base change and trinucleotide context (Supplementary Information— Methods)^6^ and mutation signature exposures were computed as the best fit to 30 reference signatures^6^ using a quadratic programming solution to non-negative least squares (R package nnls v1.4).^41^ RNA expression was analysed with respect to publically available RNA-seq tumour datasets: TCGA Research Network (http://cancergenome.nih.gov/)^5^ and Illumina Human BodyMap 2.0 RNA-seq (16 different tissues), which can be accessed from ArrayExpress (query #E-MTAB-513). Metrics calculated include expression percentile, fold change (over the mean), and kIQR (number of inter-quartile range intervals away from the median). Since there were no dataset available for porocarcinoma tumour, the TCGA average (average expression based on all TCGA cancer datasets) was used as a percentile comparator for RNA-seq data and the mean Illumina BodyMap was used for fold-change comparator. The TCGA oesophageal carcinoma (ESCA) dataset (tumour and matched normal) was also used for further comparison. Please refer to Supplementary Information for more details.

### Data availability

Whole genome sequencing and RNA-seq data (.bam files) have been submitted to the European Genome-Phenome Archive (EGA) (www.ebi.ac.uk/ega/home) under data accession number EGAD00001002596.

## Electronic supplementary material

Supplementary Information(DOCX 77 kb)

Supplementary Table S1(XLSX 8600 kb)

Supplementary Table S2(XLSX 11 kb)

Supplementary Table S3(XLSX 21 kb)

Supplementary Figure S1(PNG 631 kb)

Supplementary Figure S2(PDF 289 kb)

Supplementary Figure S3(PDF 687 kb)

Supplementary Figure S4(PDF 17 kb)

Supplementary Figure S5(PDF 7 kb)

Supplementary Figure S6(PDF 9 kb)

Supplementary Figure S7(PDF 1047 kb)

Supplementary Figure S8(PDF 4 kb)

Supplementary Figure S9(PDF 129 kb)

Supplementary Figure S10(PDF 356 kb)

## Electronic supplementary material

**Supplementary information** accompanies the paper on the *npj Precision Oncology* website (10.1038/s41698-018-0050-5).

## References

[1] Riera Leal L (2015). Eccrine porocarcinoma: epidemiologic and histopathologic characteristics. Int. J. Dermatol..

[2] Blake PW, Bradford PT, Devesa SS, Toro JR (2010). Cutaneous appendageal carcinoma incidence and survival patterns in the United States: a population-based study. Arch. Dermatol..

[3] Harms PW (2016). Porocarcinomas harbor recurrent HRAS-activating mutations and tumor suppressor inactivating mutations. Hum. Pathol..

[4] Dias-Santagata D (2011). A potential role for targeted therapy in a subset of metastasizing adnexal carcinomas. Mod. Pathol..

[5] The Cancer Genome Atlas Network. (2013). The Cancer Genome Atlas Pan-Cancer analysis project. Nat. Genet..

[6] Alexandrov LB (2013). Signatures of mutational processes in human cancer. Nature.

[7] Forbes SA (2015). COSMIC: exploring the world’s knowledge of somatic mutations in human cancer. Nucleic Acids Res..

[8] Landrum MJ (2016). ClinVar: public archive of interpretations of clinically relevant variants. Nucleic Acids Res..

[9] Martincorena I (2015). High burden and pervasive positive selection of somatic mutations in normal human skin. Science.

[10] Takata M (2000). Genetic changes in sweat gland carcinomas. J. Cutan. Pathol..

[11] Tsujita J (2015). Immunohistological expression of p16INK4a is commonly present both in benign and malignant sweat gland neoplasias. Fukuoka Igaku Zasshi.

[12] Hill VK, Gartner JJ, Samuels Y, Goldstein AM (2013). The genetics of melanoma: recent advances. Annu. Rev. Genom. Hum. Genet..

[13] Pacifico A (2008). Loss of CDKN2A and p14ARF expression occurs frequently in human nonmelanoma skin cancers. Br. J. Dermatol..

[14] Saridaki Z (2003). Mutational analysis of CDKN2A genes in patients with squamous cell carcinoma of the skin. Br. J. Dermatol..

[15] Suzuki H (1995). Intragenic mutations of CDKN2B and CDKN2A in primary human esophageal cancers. Hum. Mol. Genet..

[16] Hu N (2004). High frequency of CDKN2A alterations in esophageal squamous cell carcinoma from a high‐risk Chinese population. Genes Chromosome Cancer.

[17] Murali R, Wiesner T, Scolyer RA (2013). Tumours associated with BAP1 mutations. Pathology.

[18] Dillon LM, Miller TW (2014). Therapeutic targeting of cancers with loss of PTEN function. Curr. Drug Targets.

[19] Ming M, He YY (2009). PTEN: new insights into its regulation and function in skin cancer. J. Invest. Dermatol..

[20] Hertzler-Schaefer K (2014). Pten loss induces autocrine FGF signaling to promote skin tumorigenesis. Cell Rep..

[21] Suzuki A (2003). Keratinocyte-specific Pten deficiency results in epidermal hyperplasia, accelerated hair follicle morphogenesis and tumor formation. Cancer Res..

[22] Schneider MR, Wolf E (2009). The epidermal growth factor receptor ligands at a glance. J. Cell. Physiol..

[23] Chen J (2016). Expression and function of the epidermal growth factor receptor in physiology and disease. Physiol. Rev..

[24] Gotoh N (2009). Feedback inhibitors of the epidermal growth factor receptor signaling pathways. Int. J. Biochem. Cell. Biol..

[25] Sette G (2015). Tyr1068-phosphorylated epidermal growth factor receptor (EGFR) predicts cancer stem cell targeting by erlotinib in preclinical models of wild-type EGFR lung cancer. Cell Death Dis..

[26] Al-Rawi N, Ghazi A, Merza M (2014). PIK3CB and K-ras in oral squamous cell carcinoma. A possible cross-talk!. J. Orofac. Sci..

[27] Jamshidi F (2014). Diagnostic value of next-generation sequencing in an unusual sphenoid tumor. Oncologist.

[28] Thibodeau, M. L. et al. Genomic profiling of pelvic genital type leiomyosarcoma in a woman with a germline CHEK2:c.1100delC mutation and a concomitant diagnosis of metastatic invasive ductal breast carcinoma. *Cold Spring Harb. Mol. Case Stud*. mcs.a001628 (2017). 10.1101/mcs.a001628. 10.1101/mcs.a001628 PMC5593158 28514723

[29] Li, H. Aligning sequence reads, clone sequences and assembly contigs with BWA-MEM. (2013). Preprint at https://arxiv.org/abs/1303.3997..

[30] Li H (2009). The Sequence Alignment/Map format and SAMtools. Bioinformatics.

[31] Sheffield BS (2016). Investigation of PD-L1 biomarker testing methods for PD-1 axis inhibition in non-squamous non-small cell lung cancer. J. Histochem. Cytochem..

[32] Jones SJ (2010). Evolution of an adenocarcinoma in response to selection by targeted kinase inhibitors. Genome Biol..

[33] Ha G (2012). Integrative analysis of genome-wide loss of heterozygosity and monoallelic expression at nucleotide resolution reveals disrupted pathways in triple-negative breast cancer. Genome Res..

[34] The Cancer Genome Atlas Research Network. (2017). Integrated genomic characterization of oesophageal carcinoma. Nature.

[35] Ding J (2012). Feature-based classifiers for somatic mutation detection in tumour-normal paired sequencing data. Bioinformatics.

[36] Saunders CT (2012). Strelka: accurate somatic small-variant calling from sequenced tumor-normal sample pairs. Bioinformatics.

[37] Simpson JT (2009). ABySS: a parallel assembler for short read sequence data. Genome Res..

[38] Birol I (2009). De novo transcriptome assembly with ABySS. Bioinformatics.

[39] Robertson G (2010). De novo assembly and analysis of RNA-seq data. Nat. Methods.

[40] McPherson A (2011). deFuse: an algorithm for gene fusion discovery in tumor RNA-Seq data. PLoS Comput. Biol..

[41] Lawson, C. L. & Hanson, R. J. *Solving Least Squares Problems* 5–8 (Series: Classics in Applied Mathematics, 1995). 10.1137/1.9781611971217.ch2.

