# Supplementary material for: Whole genome and whole transcriptome genomic profiling of a metastatic eccrine porocarcinoma
Source: NPJ Precis Oncol. 2018 Mar 19;2:8. doi: 10.1038/s41698-018-0050-5 (PMC5871832; doi:10.1038/s41698-018-0050-5)
Supplement: Supplementary file 1 — Supplementary Information(DOCX 77 kb) [file 41698_2018_50_MOESM1_ESM.docx]

**SUPPLEMENTARY INFORMATION**

My Linh Thibodeau^1,2^, Melika Bonakdar^2^, Eric Zhao^2^, Karen L. Mungall^2^, Caralyn Reisle^2^, Wei Zhang^2^, Morgan H. Bye^2^, Nina Thiessen^2^, Dustin Bleile^2^, Andrew J. Mungall^2^, Yussanne P. Ma^2^, Martin R. Jones^2^, Daniel J. Renouf^3^, Howard J. Lim^3^, Stephen Yip^4^, Tony Ng^4^, Cheryl Ho^3^, Janessa Laskin^2,3^, Marco A. Marra^1,2^, Kasmintan A. Schrader*^5^, Steven J. M. Jones*^1,2^

1. Department of Medical Genetics, University of British Columbia, C201 - 4500 Oak Street, Vancouver, BC V6H 3N1 Canada, Tel 1-604-875-2157, Fax 1-604-875-2376
2. Canada's Michael Smith Genome Sciences Centre, British Columbia Cancer Agency, Vancouver, BC V5Z 4S6, Canada, Tel. 1-604-707-5800, Fax. 1-604-876-3561
3. Department of Medical Oncology, British Columbia Cancer Agency, 600 West 10th Avenue, Vancouver, BC V5Z 4E6, Tel 1-604-877-6000
4. Department of Pathology & Laboratory Medicine, Vancouver General Hospital, 910 West 10^th^ Ave, Vancouver, BC, Canada, V5Z 1M9, Tel 1-604-875-4892
5. Hereditary Cancer Program, Medical Genetics, British Columbia Cancer Agency, 614-750 West Broadway, Vancouver, BC V5Z 1H5, Canada, Tel 604-877-6000 (extension 672198)

Supplementary methods

**Tissue collection and preparation**

Lymph node biopsy tissue (10 x 3 mm) was surgically excised and embedded in optimal cutting temperature (OCT) compound and snap frozen on dry ice. 8 tubes containing 4 x 50 µm tissue sections with an estimated average tumour content of 57% (pathology review) were chosen for nucleic acid extraction using an AllPrep kit on a QiaCube instrument (Qiagen). Tumour genomic DNA (gDNA) was pooled and quantified (Qubit assay, Invitrogen), yielding 15 µg. 7 µg of total RNA was obtained with an RNA Integrity Number (RIN) of 8.1 as determined by Agilent Bioanalyzer.

**Whole genome DNA library construction**

PCR-free tumour (P00471) and blood (P00427) whole genome libraries were constructed using an automated implementation of the TruSeq DNA PCR-free kit (FC-121-1002, Illumina Inc.) from 1µg gDNA, arrayed in a 96-well microtitre plate and sheared by Covaris sonication (Perkin Elmer). Sheared DNA was end-repaired and size selected using AMPure XP beads targeting a 300-400bp fraction. After 3’ A-tailing, full length TruSeq adapters were ligated. Libraries were purified using AMPure XP beads. Library fragment sizes were assessed using an aliquot of PCR amplified library DNA on a Caliper GX DNA1000 chip. The PCR-free library concentration was quantified using a qPCR Library Quantification kit (KAPA, KK4824).

**Strand-specific RNA library construction**

A strand-specific messenger RNA library (P00475) was constructed from 1 µg total RNA. Polyadenylated (polyA+) RNA was purified using a 96-well MultiMACS mRNA isolation kit on a MultiMACS 96 separator (Miltenyi Biotec, Germany) from 1 µg total RNA with on-column DNaseI-treatment as per the manufacturer's instructions. The eluted polyA+ RNA was ethanol precipitated and resuspended in 10µL of DEPC treated water with 1:20 SuperaseIN (Life Technologies, USA). First-strand cDNA was synthesized from the purified polyadenylated messenger RNA using a Maxima H Minus First Strand cDNA Synthesis kit (Thermo-Fisher, USA) and random hexamer primers at a concentration of 5µM along with a final concentration of 1µg/uL Actinomycin D, followed by Ampure XP SPRI bead purification on a Biomek FX robot (Beckman-Coulter, USA). Second strand cDNA was synthesized following the Superscript cDNA Synthesis protocol by replacing the dTTP with dUTP in dNTP mix, allowing second strand digestion using Uracil-N-Glycosylase (Life Technologies, USA) in the post-adapter ligation reaction and thus achieving strand specificity. cDNA was fragmented by Covaris E210 sonication for 55 seconds at a “Duty cycle” of 20% and “Intensity” of 5. The paired-end sequencing library was prepared following the BC Cancer Agency Genome Sciences Centre strand-specific, plate-based and paired-end library construction protocol on a Biomek FX robot (Beckman-Coulter, USA). Briefly, the cDNA was purified in 96-well format using Ampure XP SPRI beads, and was subject to end-repair, and phosphorylation by T4 DNA polymerase, Klenow DNA Polymerase, and T4 polynucleotide kinase respectively in a single reaction, followed by cleanup using Ampure XP SPRI beads and 3’ A-tailing by Klenow fragment (3’ to 5’ exo minus). After purification using Ampure XP SPRI beads, Quant-iT quantification was performed to determine the amount of Illumina PE adapters to be used in the next step of adapter ligation reaction. The adapter-ligated products were purified using Ampure XP SPRI beads, and digested with UNG (1U/µL) at 37^o^C for 30 min followed by deactivation at 95^o^C for 15 min. The digested cDNA was purified using Ampure XP SPRI beads, and then PCR-amplified with Phusion DNA Polymerase (Thermo Fisher Scientific Inc. USA) using Illumina’s PE primer set, with cycle condition 98˚C 30sec followed by 10 cycles of 98˚C 10 sec, 65˚C 30 sec and 72˚C 30 sec, and then 72˚C 5min. The PCR products were purified using Ampure XP SPRI beads, and quality determined using a LabChip GX for DNA samples using the High Sensitivity Assay (Caliper, PerkinElmer, Inc. USA). PCR product in the 250-400bp size range was purified using SPRI beads, and the DNA quality was assessed and quantified using an Agilent DNA 1000 series II assay and Quant-iT dsDNA HS Assay Kit using Qubit fluorometer (Invitrogen), then diluted to 8nM for Illumina Sequencing.

**Whole genome and transcriptome sequencing**

Tumour and blood genome libraries were sequenced to 90X and 42X coverage, respectively using paired-end 125 base pair reads on an Illumina HiSeq2500 with version 4 chemistry. The tumour RNA library was sequenced with paired-end 75 base reads on an Illumina HiSeq2500 sequencer using version 4 chemistry, generating 186 million passed filter reads.

**Bioinformatic analysis**

*Germline alteration assessment*

Sequence reads from the whole genome libraries were aligned to the human reference genome (hg19) using Burrows-Wheeler Alignment tool (BWA-MEM v0.7.6).^1^ Variant calling and filtering was performed with mpileup and varFilter from SAMtools (v0.1.17) respectively.^2,3^

*Somatic alteration assessment*

Sequence reads from the whole genome libraries were aligned to the human reference genome (hg19) using Burrows-Wheeler Alignment tool (BWA-MEM v0.7.6).^1^ The tumor’s genomic sequence was compared to that of patient’s constitutive DNA to identify somatic alterations. Regions of copy number variation (CNV) and loss of heterozygosity (LOH) were identified using Hidden Markov model-based approaches CNAseq (v0.0.8)^4^ and APOLLOH (v0.1.2)^5^ respectively. The collection of distinct CNA and loss of heterozygosity (LOH) regions was compared to a set of theoretical models for ploidy (ranging from diploid to pentaploid) and tumour content (10% intervals from the initial estimated pathology review tumour content of 57%): 16%, 26%, 36%, 46%, 56%, 66%, 76%, 86%, 96%). The best fit was a diploid model at 66% tumour content. CNAseq (v0.0.8)^4^ and APOLLOH (v0.1.2)^6^ were used for the comparative analysis between tumour and matched normal to identify copy number variants (CNV).

and loss of heterozygosity (LOH) regions respectively.

Single nucleotide mutations were identified using a probabilistic joint variant calling approach utilizing SAMtools (v0.1.17)^2^, MutationSeq (v4.3.5)^7^ and Strelka (v1.0.6)^8^; small insertions and deletions (indels) were identified using Strelka (v1.0.6)^8^ and Trans-ABySS (v1.4.10).^9,10^ De novo assembly and annotation of genomic and transcriptomic data using ABySS (v1.3.4)^11^, Trans-ABySS (v1.4.10)^9,10^, deFuse^12^ and MAVIS (new tool, manuscript in preparation) were used to identify structural variants (SV) and fusion genes. Structural variant percentile was determined according to our local database of 584 diverse cancer cases.^13^

Variants were annotated to genes using the Ensembl database (v69).^14^ Coding single nucleotide variants (SNV) were compared to the COSMIC database (downloaded 2015/02/26)^15^ to identify previously recorded somatic events. Genes were associated with pathways and cancers using the ConsensusPathDB pathway database (v30)^16^ and the COSMIC cancer gene census (downloaded 2015/02/26).^15^ Genes were linked with potential therapeutics using DGIdb (downloaded 2016/06/21).^17^ Zygosity and variant calling of small mutations (SNV, indels) takes both the ploidy (diploid) and the tumour content (66%) into consideration.

*Transcriptome gene expression assessment*

RNA-Seq reads were aligned against a database of exon junction sequences and subsequently processed to reposition all read alignments, with gaps, onto the same genomic reference using JAGuaR (v2.2.0).^18^ Further processing (in-house software, taking into account read strand) was used to determine gene and exon read counts and normalized expression level in reads per kilobase per million mapped reads (RPKM).^19^ As no reference matched normal tissue was available for differential expression analysis, we used an approach similar to that in Jones *et al.*^4^ We compared RPKM expression levels in the tumour RNA sample to transcriptome sequencing data from 16 different normal human tissues from Illumina Human BodyMap (HBM) 2.0 project (www.illumina.com; ArrayExpress ID: E-MTAB-513)^20^, computing a fold change (FC) value compared to the appropriately matched tissue, or to the average if no appropriate match was available for the tumour sample. Gene-based RPKM (transcript-normalized) values were calculated using Ensembl (v69) gene models. To compare to TCGA datasets, which use GAF gene models, a mapping from a given Ensembl gene to the appropriate GAF gene was determined based on position overlap.

The Cancer Genome Atlas (TCGA) gene expression data (level 3) was downloaded (https://tcga-data.nci.nih.gov/tcga/) and analyzed to calculate reads per kilobases per million mapped reads (RPKM) values from each of the available cancer types. Each gene in the patient tumour sample was compared to a ranked list of all TCGA expression values for that gene to assign it an overall tumour percentile within the same tumour type if available, or within all tumour types. A within sample expression rank was also calculated for each gene to further infer significance to outlier gene expression levels. To select a subset of genes for correlation analysis, we implemented ANOVA on log-transformed TCGA expression data. Expression data for the top 3000 genes ranked by F-ratios were normalized (mean) across TCGA samples and the patient sample. We then computed Spearman correlations between TCGA expression datasets and the patient sample.

*Biological interpretation and therapeutic association*

Alterations with specific therapeutic associations are identified using the Genome Sciences Centre's expert curated Knowledgebase, which integrates information from sources including cancer databases, drug databases, clinical tests, and the biomedical literature. Associations are considered based on the level of evidence for the use of that drug in the context of the observed alteration, including those that are approved in the same or other cancer types, and those that have early clinical or preclinical evidence. Inferences about novel or poorly studied alterations are made based on the biological function and activity of the protein and the expected impact of the alteration. Additional biological and therapeutic inferences are made based on patient-specific annotated pathways. The comprehensive tumour description is expert reviewed, including highlighting driver mutations, providing pathway context, interpreting results in tumour type context, and refining potential therapeutic targets.

**Mutational signatures**

*Monte-Carlo simulation*

The mutation signature profile was determined by classifying all genomic SNVs into 96 classes based on variant and 3’/5’ mutation context to obtain a mutation catalog vector as described by Alexandrov *et al*. (2013).^21^ In order to determine the best fit to a consensus set of 30 mutation signatures (available at <http://cancer.sanger.ac.uk/cosmic/signatures>), we performed non-negative least squares decomposition to determine the exposure vector **e** in the model $\mathbf{m}=\mathbf{Se}$. **S** is known 96x30 matrix of consensus signatures, **m** is the 96-element mutation catalog, and the members of **e** denote each signature’s relative contribution to the overall mutation burden. Determining the solution of **e** which minimizes the squared residuals, $\mathrm{argmin}_{\mathbf{e}} \left\| \mathbf{Se-m} \right\|^{2}$, is a well-studied problem. We determined **e** using a quadratic programming approach implemented in the R package “nnls” (version 1.4) which rapidly converges to an optimal solution. In order to estimate the sampling variance inherent in **m**, we performed Monte Carlo simulation. 1000 replicate vectors, $\mathbf{m}_{i}^{*}, i=1, 2, \ldots1000$, were randomly drawn from a multinomial distribution, $\mathbf{m}_{i}^{*}\boldsymbol{\sim}Multinomial(n, p= \frac{\mathbf{m}}{n})$, where *n* is the number of somatic mutations. 1000 corresponding exposure vectors were computed from the simulated catalogs and the 95% simulated interval was reported using the 25^th^ and 975^th^ ranked values for each signature exposure.

*Timing of mutational processes*

Mutations were partitioned into early and late categories as described by McGranahan *et al* (2015).^22^ Early mutations represent clonal variants or those present in multiple copies in a region with copy number gain (and which therefore likely occurred before the duplication). Late mutations represent subclonal variants which were not present prior to copy number gains. Mutation signatures were deciphered in the previously described manner for both early and late variants.

*Radiation exposure analysis*

Indel/substitution and deletion/insertion ratios were analyzed as a surrogate of radiation-induced mutational processes as described by Behjati *et al* (2016), who found that radiation-induced tumours had higher such ratios.^23^ A custom analysis was carried out, which included genomewide assessment of indels and SNV, detection of microhomology fraction and fraction of deletions in simple repeat regions.

Supplementary clinical description

The male patient presented at the age of 64 with a bleeding left-scalp lesion. The surgical excision biopsy revealed a well-circumscribed, but atypical proliferation of squamoid cells with connection to the overlying epidermis and conspicuous mitotic activity (10-15 mitoses per 10 high power fields) with foci of necrosis. The lesion was resected and pathology examination revealed clear margins. Pathology examination showed features in keeping with eccrine porocarcinoma (EP). Eighteen months later, the patient presented with left cervical lymphadenopathies. Neck ultrasound imaging showed numerous pathological-appearing cervical lymph nodes, the largest measuring 1.2x1.2x1.2 cm. A fine-needle aspiration did not yield a satisfactory pathological sample, and was followed by a 2.5 cm incisional biopsy of a lymph node next to the left sternocleidomastoid muscle. Tumour staging investigations included an FDG-PET scan, showing the left occipital malignancy and incidentally identifying a pituitary adenoma, which maintained stable appearance on subsequent imaging. Oncological management included left neck radical dissection with en bloc excision of the left occipital porocarcinoma mass, followed by local radiation therapy to the occipital, cervical and supraclavicular areas (60 Gy in 30 fractions). Post-treatment PET scan imaging was negative for evidence of distant metastasis. Over a three month-period following initial management completion, the patient developed increasing pain in the left neck and left shoulder area, but CT and FDG-PET scan imaging did not uncover any suspicious focus of malignancy. Six months later, the follow-up FDG-PET scan showed a tracer avid left supraclavicular node and fine needle aspiration cytology revealed features in keeping with metastatic carcinoma. Subsequent wide incisional biopsy of a posterior triangle cervical lymph node confirmed the malignant epithelial neoplasm consistent with metastatic EP. Systemic therapies were considered, but not pursued, given the patient's desire to optimize his quality of life and the lack of scientific literature supporting efficacy of such therapies in EP management. Shortly after, the patient acutely developed cerebellar signs with pronounced slurred speech and truncal ataxia. No acute vascular event was seen on unenhanced brain CT and contrast CT-angiography imaging, but brain MRI with gadolinium showed an enhancing mass (1.9x3.3x2.1 cm) within the superior vermis and right superior cerebellar hemisphere. Right occipital craniotomy and cerebellar metastasis surgical resection was performed for symptomatic relief. Pathology of the resected tissue confirmed metastatic EP (Fig. 1).

The patient developed leptomeningeal disease over the following two months and passed away from progressive central nervous system involvement.

Supplementary results and discussion

**Somatic genomic variants**

Please refer to Supplementary Table S1, Table S2 and Table S3 for comprehensive results. Supplementary Table S1A presents a summary of somatic mutational burden, including single nucleotide variants (SNV), indels and structural variants (SV). We also present the Spearman correlation comparing RNAseq transcriptome data from our patient and TCGA expression datasets, which showed the highest correlation with the TCGA esophageal carcinoma (ESCA) dataset (Supplementary Fig. S3).^24,25^

Even though a copy loss of *CTNNB1* gene (coding β-catenin protein) was present, gene expression was not obviously perturbed (Table 1) and tumour sample IHC staining for β-catenin did not show nuclear translocation (Fig. 1e). *APC* LOH, which was found in our tumour, is a relatively frequent somatic event in eccrine poromas and porocarcinomas.^26^ Germline predisposition to benign eccrine poromas has been described in a rare ectodermal dysplasia: Schöpf-Schulz-Passarge syndrome (SSPS) caused by *WNT10A* mutations (MIM #224750). *WNT10A* was markedly overexpressed (Supplementary Table S1B), and future research may uncover a role for WNT10A ligand in eccrine porocarcinoma pathogenesis.

We observed a somatic *PIK3CB* copy gain (4 copies) in our patient's tumour and *PIK3CB* activity in PTEN-deficient cancer cells promotes growth and proliferation.^27^ Recently, *KRAS* and *PIK3CB* signaling have been noted to have a direct relationship in oral squamous cell carcinomas and these oncogenes may become therapeutic targets in the future.^28^ *RB1* had elevated expression when compared to other squamous cell carcinomas (Table 1).

Although gene expression of collapsed transcripts (all isoforms included) was similar to other TCGA datasets (Table 1), further analysis of transcript-specific expression revealed that the *CDKN2A* p16^INK4a^ isoform had relatively low RPKM expression and p14^ARF^ isoform had overall average expression (Supplementary Results Table S1, Table S2, Fig. S7 and Fig. S8).

*Structural variants*

***RNF13*-*PAK2* fusion**

A 46Mb deletion on chromosome 3 (chr3:149653091-196530353, hg19, Supplementary Table S1D, Fig. S9) leads to the fusion of *RNF13* (intron 9 breakpoint) and *PAK2* (intron 4 breakpoint). This genomic event is well supported in the transcriptome. This event leads to loss of RNF13 RING domain, and as RNF13 is thought to be a ubiquitin ligase, its RING domain would be critical to this functionality^29^ and loss of function can be inferred from this genomic event. Although RNF13 activity was initially thought to favor cancer progression,^29^ reduced RNF13 has also been shown to enhance metastasis.^30^ The role of this gene in cancer pathogenesis remains unclear. Due to the genomic event, *PAK2* loses its p21-RHO-binding domain, which is the required for binding of CDC42 and RAC1 and subsequent increases of PAK2 kinase activity. However, as PAK2 loses its GTPase-binding domain, necessary for dimer and autoinhibition, this deletion event could lead to constitutive activation of this serine threonine kinase receptor. *PAK2* has a two copy gain (total of 4 copies) and its expression is increased (100^th^ percentile among ESCA TCGA cancers and 99^th^ percentile among all TCGA cancers). Transcriptome data revealed the expression of a chimeric transcript fusing the first eight exons of *RNF13* to exons 5 to 15 of *PAK2*. Transcriptome support for the event is high (more than 500 flanking read pairs, 386 spanning reads of which 203 are forward reads and 183 are reverse reads). *PAK2* was shown to play a role in multiple cancer types including ovarian, endometrial, breast and others.^31-36^ Taken together with the loss of *PAK2* GTPase-binding domain, the copy gain (4 copies in total) and high expression of PAK2 support a potential role for PAK2 in our patient’s EP oncogenesis, but a functional assay (e.g. phosphorylation assay) would be required to investigate further this hypothesis.

***PIK3R1*-*YTHDC2* fusion**

A 45Mb deletion on chromosome 5 (chr5:67564688-112859542, hg19, Supplementary Table S1D, Fig. S10) leads to the fusion of *PIK3R1* (intron 2 breakpoint) and *YTHDC2* (intron 2 breakpoint). All critical *YTHDC2* domains (RNA helicase related) are maintained, but the ssDNA/RNA binding domain (R3H) is lost due to the fusion and therefore, loss of function is inferred from the absence of binding domain R3H. The functional domain of *PIK3R1* (encoded by exon 2) is required for interaction with adaptor proteins and tyrosine kinases and it is maintained. Transcriptome data revealed the expression of a chimeric transcript fusing the first two exons of *PIK3R1* to exons 4 to 17 of *YTHCD2*. Transcriptome support for the event is high (37 flanking read pairs, 32 spanning reads of which 18 are forward reads and 12 are reverse reads). *PIK3R1* showed increased expression (96^th^ percentile among ESCA TCGA cancers, 87^th^ percentile among all TCGA cancers) while *YTHDC2* expression slightly elevated (73^rd^ percentile among ESCA TCGA cancers, 85^th^ percentile among all TCGA cancers). While *PIK3R1* is thought to have tumour suppressor properties by negatively regulating the epithelial-to-mesenchymal transition in renal cancer cells via the AKT/GS3KB/CTNNB1 pathway,^37^ some data suggests that *YTHDC2* may act as an oncogene by enhancing the efficiency of *HNF1A* translational process. *HNF1A* expression level was unremarkable (Supplementary Table S1B). Therefore, the biological relevance of this structural variants remains unclear.

**Mutational signatures**

*Monte-Carlo simulation*

The somatic SNV profile revealed a best-fit mutation signature model comprised of signatures 1, 8, 9, and 16 (Supplementary Table S3A, Fig. S4). Signature 1 is associated with age, and is ubiquitous across cancer types. Signature 9 is attributed to polymerase η mutagenesis.^38^ The etiologies of signatures 8 and 16 are not clearly characterized. While Signature 8 has been weakly associated with homologous recombination deficiency, the role of the more specific Signature 3 was unclear in this tumour.^39^

*Timing of mutational processes*

Using techniques described by McGranahan *et al* (2015)^22^, we can partition mutations into likely early and likely late, and then decipher mutation signatures separately for both (Supplementary Table S3B-C, Fig. S5).

Temporal dissection of mutations revealed 1469 late-arising and subclonal variants and 2605 early- arising or clonal variants. Mutation signatures deciphered from temporally dissected SNVs demonstrated stable representation of signatures 8, 9, and 16 across early and late mutations. Signature 1 decreased over time, agreeing with previously reported findings by McGranahan *et al* (2015). Late mutations also included newly arising signatures 27 (unknown etiology) and 30 (associated with *NTHL1* mutations).^40^ Contrary to previously reported porocarcinoma cases, signatures of UV light exposure were not elevated in either early or late mutations.

*Radiation exposure analysis*

Using techniques described by Behjati *et al* (2016)^23^, we did not identify a clear pattern of radiation-induced mutational processes. We note that the overall analysis of potential radiation-induced genomic features (Supplementary Table S3D) is inconsistent and therefore inconclusive. Although the fraction of deletions in simple repeat regions is 4.2%, a number above what could be expected by chance given the fact that simple repeats cover only 2.5% of the hg19 genome, the distribution of deletions across the genome display variation, with distances between deletions ranging from 100 kb to 100 Mb (Supplementary Fig. S6), which is not in keeping with the equal distribution radiation-induced deletions described by Behjati *et al* (2016).^23^ The deletion microhomology fraction (8.6%) was low and we did not identify an increase in large inversion SV, which also does not support radiation-induced processes either.^23^

Although Behjati *et al* (2016)^23^ looked at inferred radiation-induced tumours rather than post-radiation treatment tumours, tumour exposed to radiotherapy could theoretically present similar genomic features. Our EP’s indels and substitution SNV features did not point towards radiation-associated processes.

Temporal analysis of mutational signatures (Supplementary Fig. S5) shows Signature 30 as a late-arising mutational signature. Signature 30 has been associated with *NTHL1* mutations. ^40^ We can not comment to whether radiotherapy in our patient played a role in porocarcinoma mutational signatures as a whole.^21,23^

Signature 7 is associated with UV-induced mutagenesis.^21^ The NNLS score of Signature 7 was 0.015, suggesting that up to 1.5% of mutational processes may be attributable to UV-related processes, but the confidence interval of this signature crosses zero in the Monte Carlo simulation, therefore Signature 7 is unlikely a significant contributor to the porocarcinoma tumour of our patient.

Supplementary Tables

**Supplementary Table S1.** (S1A) mutation summary (S1B) copy number and RNA expression data (S1C) small mutations (coding single nucleotide variants and indels) (S1D) structural variants and (S1E) sequencing summary.

**Supplementary Table S2.** (S2A) *CDKN2A* transcripts coverage and (S2B) *CDKN2A* exon coverage-merged.

**Supplementary Table S3.** (S3A) Signatures Monte-Carlo (S3B) Signatures timing Early - as described by McGranahan *et al* (2015) (S3C) Signatures timing Late - as described by McGranahan *et al* (2015) (S3D) Potential radiation-induced mutational features as described by Behjati *et al* (2016).

**Supplementary Table S4.** Somatic copy number variants (CNVs) – Number of hugo genes involved

| Copy number variants | Number of genes involved in CNV events |
| --- | --- |
| **Copy neutral (HET)** | 8198 |
| **LOSS** | |
| DLOH (deletional LOH) | 1073  (5 homozygous loss: *LRRC31*, *GPB3*, *AMY1B*, *AMY1A*, *AMY2A*) |
| NLOH (copy neutral LOH) | 564 |
| **GAIN** | |
| ASCNA | 160 |

Supplementary figures legends

**Supplementary Figure S1.** CIRCOS plot^41^ illustrating the somatic copy number variants (CNV) observed in the porocarcinoma tumour. From the outer circle: copy gains are represented in red; copy losses are represented in green and region of loss of heterozygosity (LOH) are represented in blue.

**Supplementary Figure S2.** Copy number and loss of heterozygosity (LOH) for chromosome 1, 3, 5, 6 and 9. Chromosome 1 has a large copy loss involving centromeric half of p-arm. Chromosome 3 has a large copy loss involving p-arm and q-arm, and a copy gain on q-arm. Chromosome 5 has a large copy loss involving q-arm. Chromosome 6 has some focal copy losses. Chromosome 9 has some focal deletional copy loss at p22.2-p21.3 encompassing *CDKN2A, CDKN2B, CDKN1A.*

**Supplementary Figure S3.** Transcriptome Spearman correlation across TCGA cancer types. The highest correlation found was with squamous cancers, specifically esophageal squamous carcinoma (ESCA). ACC, adrenocortical carcinoma; BLCA, bladder urothelial carcinoma; BM, bonne marrow; BRCA, breast invasive cancer; CESC CAD, cervical squamous cell carcinoma and endocervical adenocarcinoma - cervical adenocarcinoma; CESC SCC, cervical squamous cell carcinoma and endocervical adenocarcinoma - squamous cell carcinoma; CHOL, cholangiocarcinoma; COADREAD, colorectal adenocarcinoma; DLBC, diffuse large B-cell lymphoma; DLBCL, diffuse large B-cell lymphoma; ESCA EAC, esophageal carcinoma esophageal adenocarcinoma; ESCA SCC, esophageal carcinoma squamous cell carcinoma; FL, follicular lymphoma; GBM, glioblastoma; HNSC, head and neck squamous cell carcinoma; KICH, kidney chromophobe; KIRC, kidney renal clear cell carcinoma; KIRP, kidney renal papillary cell carcinoma; LAML, acute myeloid leukemia; LGG, low grade glioma; LIHC, liver hepatocellular carcinoma; LUAD, lung adenocarcinoma; LUSC, lung adenocarcinoma; MB, medulloblastoma; MESO, mesothelioma; NCI, National Cancer Institute; OV, ovarian serous cystadenocarcinoma; PAAD, pancreatic adenocarcinoma; PCPG, pheochromocytoma and paraganglioma; PRAD, prostate adenocarcinoma; SARC, sarcoma; SKCM, skin cutaneous melanoma; STAD, stomach adenocarcinoma; TFRI, Terry Fox Research Institute; TGCT, testicular germ cell tumors; THCA, thyroid carcinoma; THYM, thymoma; UCEC, uterine corpus endometrial carcinoma; UCS, uterine carcinosarcoma; UVM, uveal melanoma.

**Supplementary Figure S4.** Mutational signatures – Monte-Carlo simulation. Exposure (mutation count) for each mutational signature. The exposure is displayed on the x-axis and represents the total number of mutations contributing to each individual signature on the y-axis. Non-negative least squares (NNLS) with Monte Carlo resampling was used to generate 95% confidence interval for the mutation count of each signature.

**Supplementary Figure S5.** Mutational signatures timing as described by McGranahan *et al* (2015).^22^ (A) Early mutations (B) Late mutations.

**Supplementary Figure S6.** Genomewide deletion distribution as described by Behjati *et al* (2016).^23^

**Supplementary Figure S7.** *CDKN2A* transcripts (green/blue) and expression overlay (top). The depth of read coverage in the transcriptome is given by the top graph. Ensembl transcript diagrams are depicted below where IDs are given to the left of each transcript and exonic regions have been scaled larger than intronic regions. The position of the splice-site mutation (9:21971208C>T) is noted with the vertical line running perpendicular to the transcripts.

**Supplementary Figure S8.** Exon-specific collapsed transcripts expression of *CDKN2A* transcripts NM_058195 (p14ARF) and NM_000077 (p16INK4a). RPKM (reads per kilobase of transcript per million mapped reads) coverage on the Y-axis in relation to genomic regions approximately corresponding to *CDKN2A* exons on X-axis (Exon 1 p14ARF chr9:21994138-21994490; Exon 1 p16INK4a chr9:21970901-21971207; Exon 2 p14ARF/p16INK4a chr9: 21967752-21968241; Exon 3 p14ARF/p16INK4a chr9:21968574-21968770; hg19).

**Supplementary Figure S9.** Large 46Mb chromosome 3 deletion (3:149653091-196530353, hg19) creating a *RNF13*-*PAK2* gene fusion expressed in the transcriptome. This event leads to loss of RNF13 RING domain, which is predicted to be essential for this ubiquitin ligase. *PAK2* loses its p21-RHO-binding domain, which is the required for binding of CDC42 and RAC1 and subsequent increases of PAK2 kinase activity. As *PAK2* loses its GTPase-binding domain, which is necessary for dimer and autoinhibition, this deletion event could potentially lead to constitutive activation of this serine threonine kinase receptor. *PAK2* has a two-copy gain (total of 4 copies) and its expression is increased (99^th^ percentile). Given the role of *PAK2* in cancer progression, these genomic events potentially play a role the pathogenesis of our patient’s EP.

**Supplementary Figure S10.** Large 45Mb chromosome 5 deletion (chr5:67564688-112859542, hg19) creating a *PIK3R1*-*YTHDC2* gene fusion expressed in the transcriptome. All critical *YTHDC2* domains (RNA helicase related) are maintained, but the ssDNA/RNA binding domain (R3H) is lost. The functional domain of PIK3R1 (encoded by exon 2) is required for interaction with adaptor proteins and tyrosine kinases and it is maintained. The expression of *PIK3R1* is high (96^th^ percentile), and this gene has both tumour suppressor and oncogene properties.

SUPPLEMENTARY REFERENCES

1. Li, H. Aligning sequence reads, clone sequences and assembly contigs with BWA-MEM. *arXiv preprints.* **00,** 1–3 (2013).

2. Li, H. *et al.* The Sequence Alignment/Map format and SAMtools. *Bioinformatics.* **25,** 2078–2079 (2009).

3. Sheffield, B. S. *et al.* Investigation of PD-L1 Biomarker Testing Methods for PD-1 Axis Inhibition in Non-squamous Non-small Cell Lung Cancer. *J. Histochem. Cytochem.* **64,** 587–600 (2016).

4. Jones, S. J. *et al.* Evolution of an adenocarcinoma in response to selection by targeted kinase inhibitors. *Genome Biol.* **11,** R82 (2010).

5. The Cancer Genome Atlas Research Network*.* Integrated genomic characterization of oesophageal carcinoma. *Nature.* **541,** 169–175 (2017).

6. Ha, G. *et al.* Integrative analysis of genome-wide loss of heterozygosity and monoallelic expression at nucleotide resolution reveals disrupted pathways in triple-negative breast cancer. *Genome Research.* **22,** 1995–2007 (2012).

7. Ding, J. *et al.* Feature-based classifiers for somatic mutation detection in tumour-normal paired sequencing data. *Bioinformatics.* **28,** 167–175 (2012).

8. Saunders, C. T. *et al.* Strelka: accurate somatic small-variant calling from sequenced tumor-normal sample pairs. *Bioinformatics.* **28,** 1811–1817 (2012).

9. Simpson, J. T. *et al.* ABySS: A parallel assembler for short read sequence data. *Genome Research.* **19,** 1117–1123 (2009).

10. Birol, I. *et al.* De novo transcriptome assembly with ABySS. *Bioinformatics.* **25,** 2872–2877 (2009).

11. Robertson, G. *et al.* De novo assembly and analysis of RNA-seq data. *Nature Methods.* **7,** 909–912 (2010).

12. McPherson, A. *et al.* deFuse: An Algorithm for Gene Fusion Discovery in Tumor RNA-Seq Data. *PLOS Comput. Biol.* **7,** e1001138–16 (2011).

13. Thibodeau, M. L. *et al.* Genomic profiling of pelvic genital type leiomyosarcoma in a woman with a germline CHEK2:c.1100delC mutation and a concomitant diagnosis of metastatic invasive ductal breast carcinoma. *Cold Spring Harb. Mol. Case. Stud.* mcs.a001628 (2017). doi:10.1101/mcs.a001628

14. Flicek, P. *et al.* Ensembl 2014. *Nucleic Acids Res.* **42,** D749–55 (2014).

15. Forbes, S. A. *et al.* COSMIC: mining complete cancer genomes in the Catalogue of Somatic Mutations in Cancer. *Nucleic Acids Res.* **39,** D945–50 (2011).

16. Herwig, R., Hardt, C., Lienhard, M. & Kamburov, A. Analyzing and interpreting genome data at the network level with ConsensusPathDB. *Nature Protocols.* **11,** 1889–1907 (2016).

17. Wagner, A. H. *et al.* DGIdb 2.0: mining clinically relevant drug-gene interactions. *Nucleic Acids Res.* **44,** D1036–44 (2016).

18. Butterfield, Y. S. *et al.* JAGuaR: junction alignments to genome for RNA-seq reads. *PLOS One*. **9,** e102398 (2014).

19. Mortazavi, A., Williams, B. A., McCue, K., Schaeffer, L. & Wold, B. Mapping and quantifying mammalian transcriptomes by RNA-Seq. *Nature Methods.* **5,** 621–628 (2008).

20. Asmann, Y. W. *et al.* Detection of redundant fusion transcripts as biomarkers or disease-specific therapeutic targets in breast cancer. *Cancer Res.* **72,** 1921–1928 (2012).

21. Alexandrov, L. B., Nik-Zainal, S., Wedge, D. C., Campbell, P. J. & Stratton, M. R. Deciphering Signatures of Mutational Processes Operative in Human Cancer. *Cell Rep.* **3,** 246–259 (2013).

22. McGranahan, N. *et al.* Clonal status of actionable driver events and the timing of mutational processes in cancer evolution. *Sci. Transl. Med.* **7,** 283ra54–283ra54 (2015).

23. Behjati, S. *et al.* Mutational signatures of ionizing radiation in second malignancies. *Nat. Commun.* **7,** 12605 (2016).

24. Lim, E. L. *et al.* Comprehensive miRNA sequence analysis reveals survival differences in diffuse large B-cell lymphoma patients. *Genome Biol.* **16,** 18 (2015).

25. Altman, N. & Krzywinski, M. Points of Significance: Association, correlation and causation. *Nature Methods.* **12,** 899–900 (2015).

26. Ichihashi, N. & Kitajima, Y. Loss of heterozygosity of adenomatous polyposis coli gene in cutaneous tumors as determined by using polymerase chain reaction and paraffin section preparations. *J. Dermatol. Sci.* **22,** 102–106 (2000).

27. Wee, S. *et al.* PTEN-deficient cancers depend on PIK3CB. *Proc. Natl. Acad. Sci. USA.* **105,** 13057–13062 (2008).

28. Al-Rawi, N., Ghazi, A. & Merza, M. PIK3CB and K-ras in oral squamous Cell carcinoma. A possible cross-talk! *J. Orofac. Sci.* **6,** 99–5 (2014).

29. Zhang, Q., Meng, Y., Zhang, L., Chen, J. & Zhu, D. RNF13: a novel RING-type ubiquitin ligase over-expressed in pancreatic cancer. *Cell Res.* **19,** 348–357 (2009).

30. Cheng, H., Wang, A., Meng, J., Zhang, Y. & Zhu, D. Enhanced metastasis in RNF13 knockout mice is mediated by a reduction in GM-CSF levels. *Protein & Cell.* **6,** 746–756 (2015).

31. Stofega, M. R., Sanders, L. C., Gardiner, E. M. & Bokoch, G. M. Constitutive p21-activated kinase (PAK) activation in breast cancer cells as a result of mislocalization of PAK to focal adhesions. *Mol. Biol. Cell.* **15,** 2965–2977 (2004).

32. Siu, M. K. Y. *et al.* Differential expression and phosphorylation of Pak1 and Pak2 in ovarian cancer: effects on prognosis and cell invasion. *Int. J. Cancer.* **127,** 21–31 (2009).

33. Flate, E. & Stalvey, J. R. D. Motility of select ovarian cancer cell lines: Effect of extracellular matrix proteins and the involvement of PAK2. *Int. J. Oncol.* **45,** 1401–1411 (2014).

34. Marlin, J. W., Eaton, A., Montano, G. T., Chang, Y.-W. E. & Jakobi, R. Elevated p21-Activated Kinase 2 Activity Results in Anchorage-Independent Growth and Resistance to Anticancer Drug–Induced Cell Death. *Neoplasia.* **11,** 286–297 (2009).

35. Siu, M. K. Y. *et al.* p21-Activated Kinases 1, 2 and 4 in Endometrial Cancers: Effects on Clinical Outcomes and Cell Proliferation. *PLOS ONE.* **10,** e0133467–13 (2015).

36. Radu, M., Semenova, G., Kosoff, R. & Chernoff, J. PAK signalling during the development and progression of cancer. *Nat. Rev. Cancer.* **14,** 13–25 (2014).

37. Lin, Y. *et al.* PIK3R1 negatively regulates the epithelial-mesenchymal transition and stem-like phenotype of renal cancer cells through the AKT/GSK3β/CTNNB1 signaling pathway. *Sci. Rep.* **5,** 8997–12 (2015).

38. Alexandrov, L. B. *et al.* Signatures of mutational processes in human cancer. *Nature.* **500,** 415–421 (2013).

39. Davies, H. *et al.* HRDetect is a predictor of BRCA1 and BRCA2 deficiency based on mutational signatures. *Nat. Med.* **23,** 517–525 (2017).

40. Drost, J. *et al.* Use of CRISPR-modified human stem cell organoids to study the origin of mutational signatures in cancer. *Science.* **358,** 234–238 (2017).

41. Krzywinski, M. *et al.* Circos: an information aesthetic for comparative genomics. *Genome Research.* **19,** 1639–1645 (2009).
