# Supplementary figures and images for: Whole genome and whole transcriptome genomic profiling of a metastatic eccrine porocarcinoma
Source: NPJ Precis Oncol. 2018 Mar 19;2:8. doi: 10.1038/s41698-018-0050-5 (PMC5871832; doi:10.1038/s41698-018-0050-5)

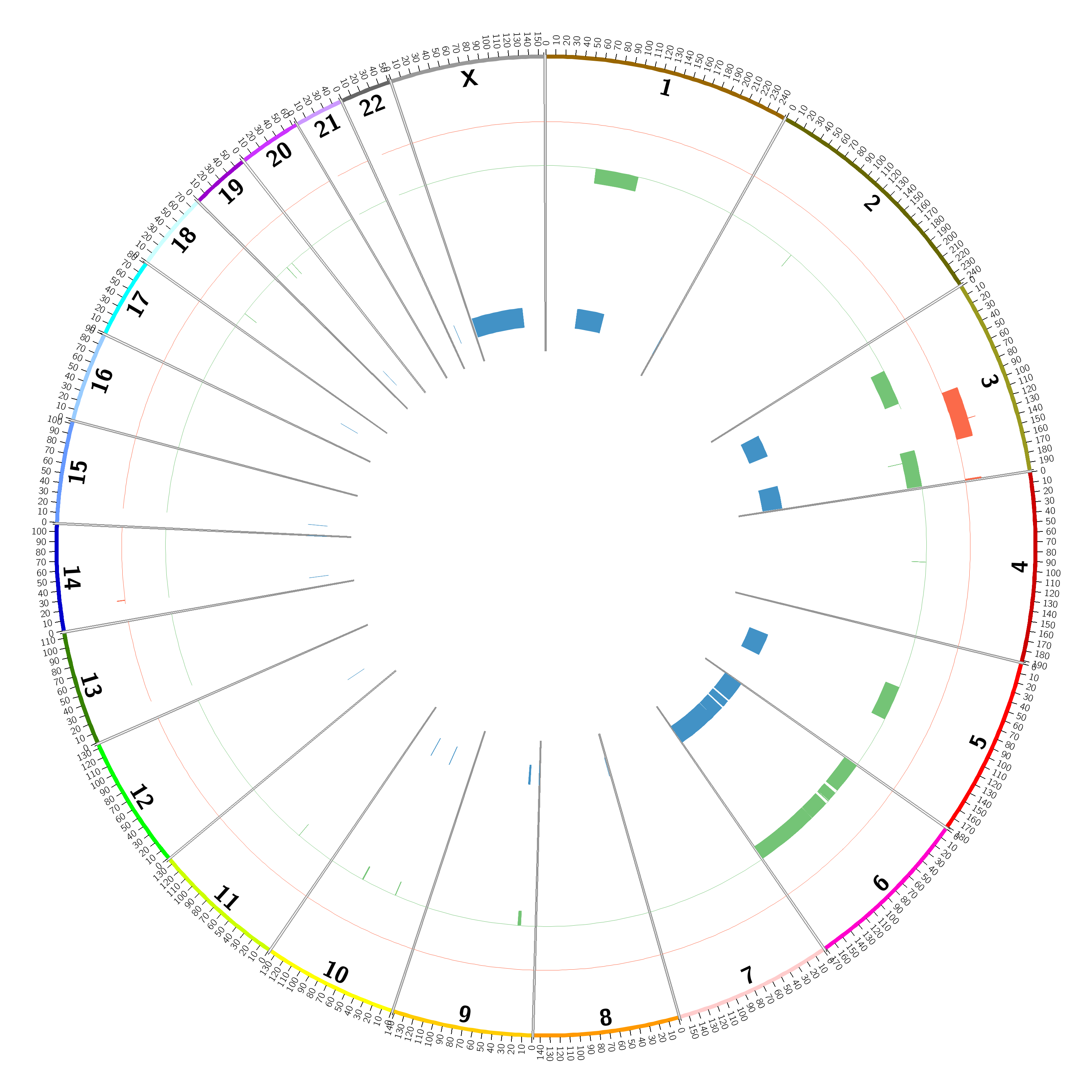

Supplement: Supplementary file 5 — Supplementary Figure S1(PNG 631 kb) [file 41698_2018_50_MOESM5_ESM.png]

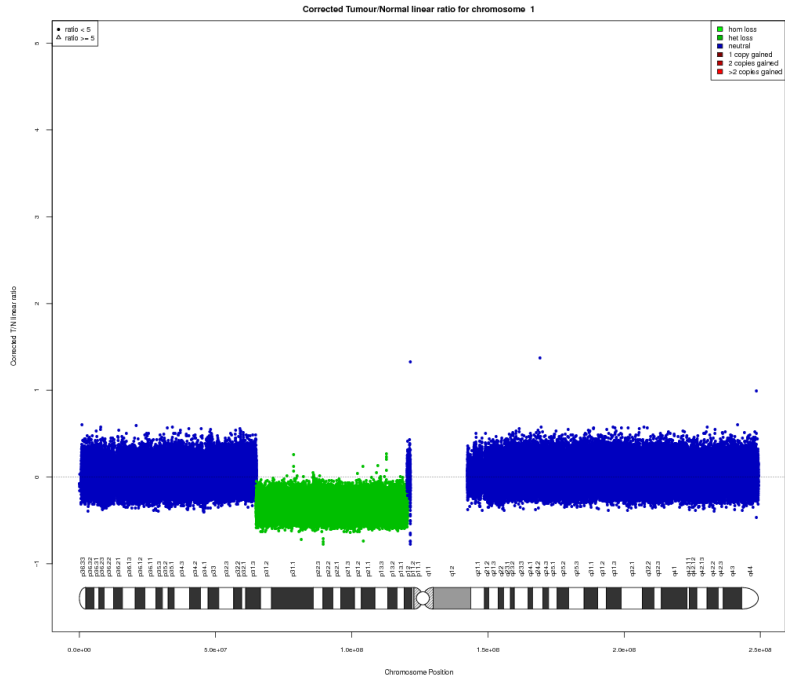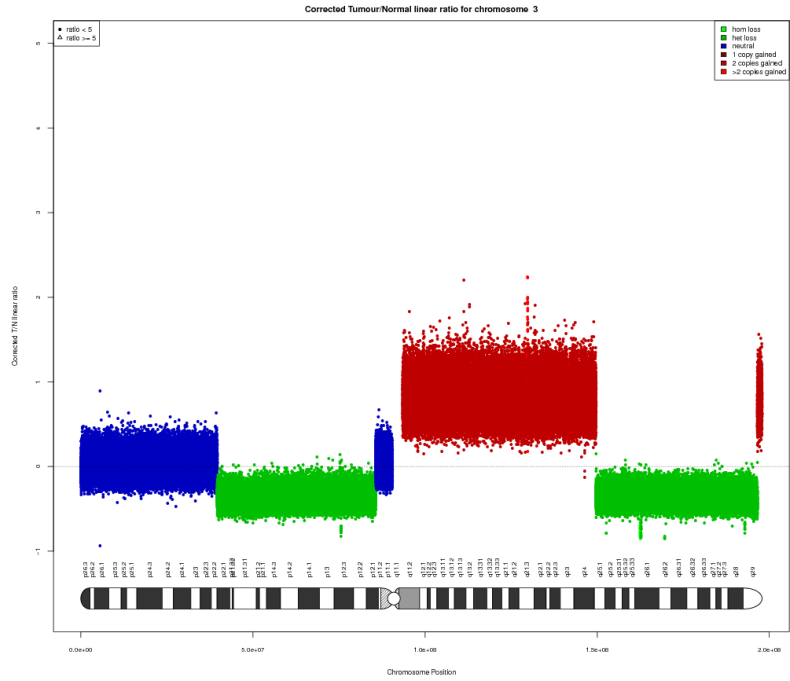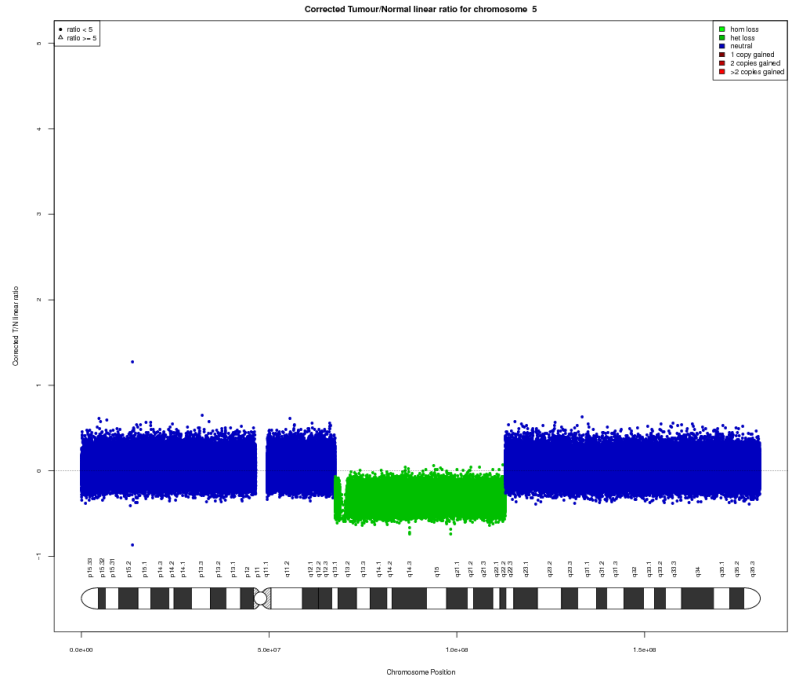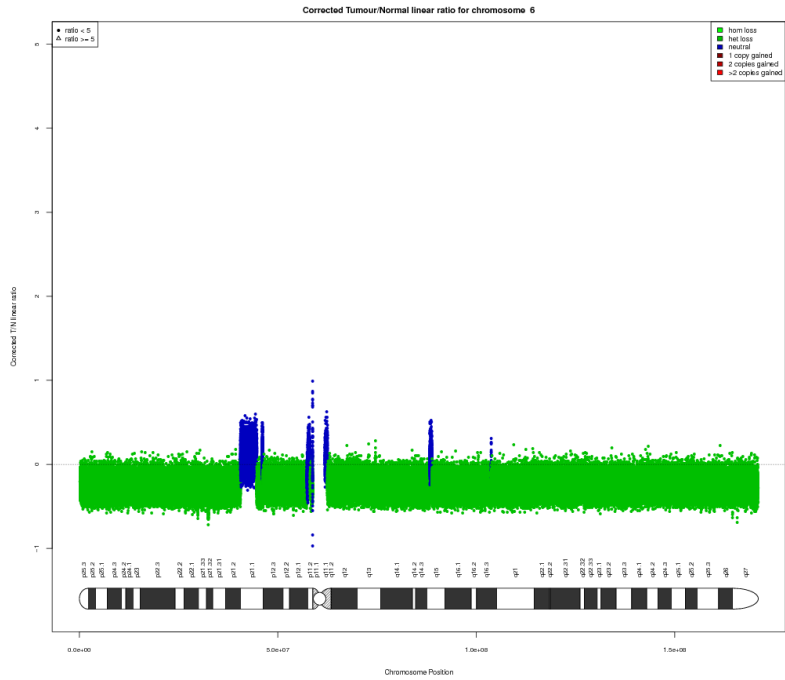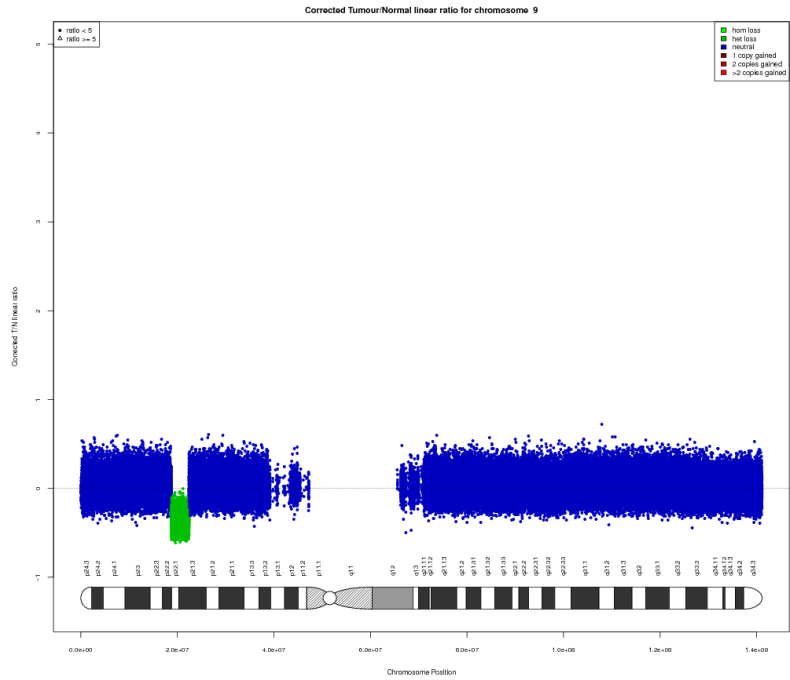

Supplement: Supplementary file 6 — Supplementary Figure S2(PDF 289 kb) [file 41698_2018_50_MOESM6_ESM.pdf]

**A****Porocarcinoma Case**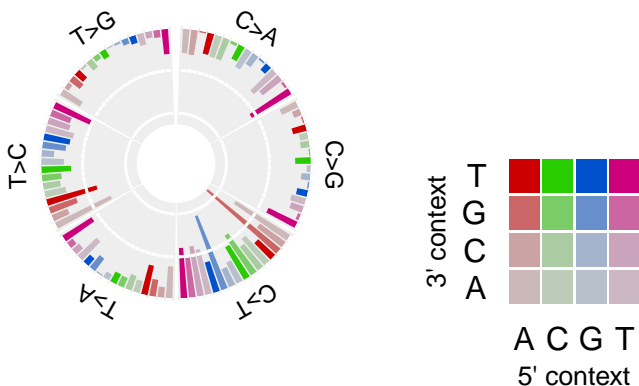**B**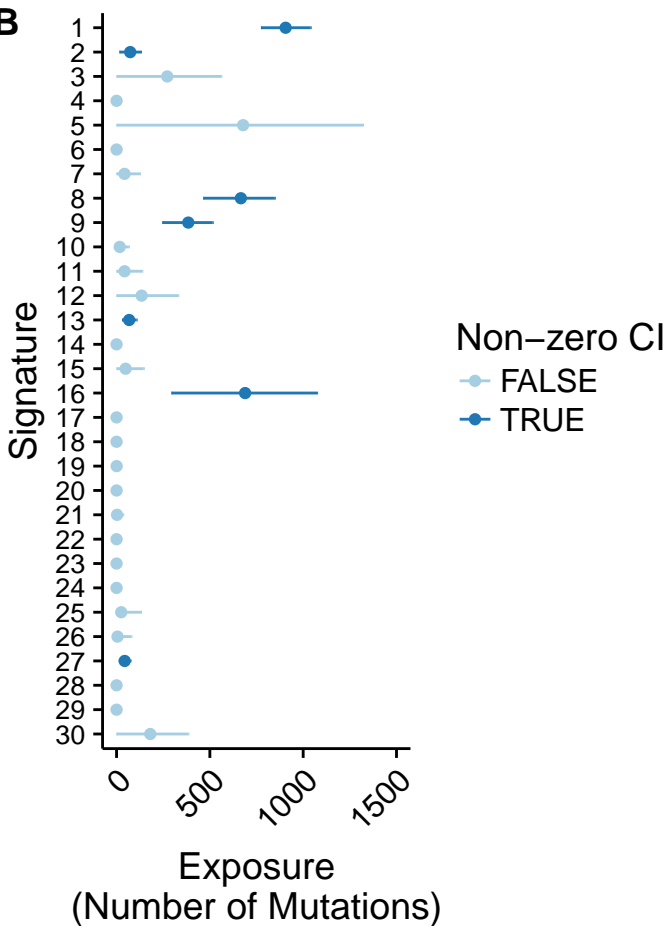

Supplement: Supplementary file 8 — Supplementary Figure S4(PDF 17 kb) [file 41698_2018_50_MOESM8_ESM.pdf]

## Early Mutations

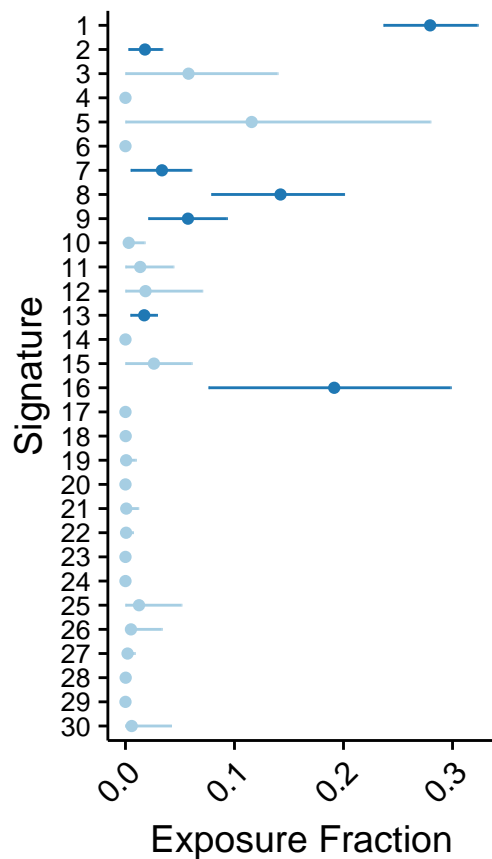

## Late Mutations

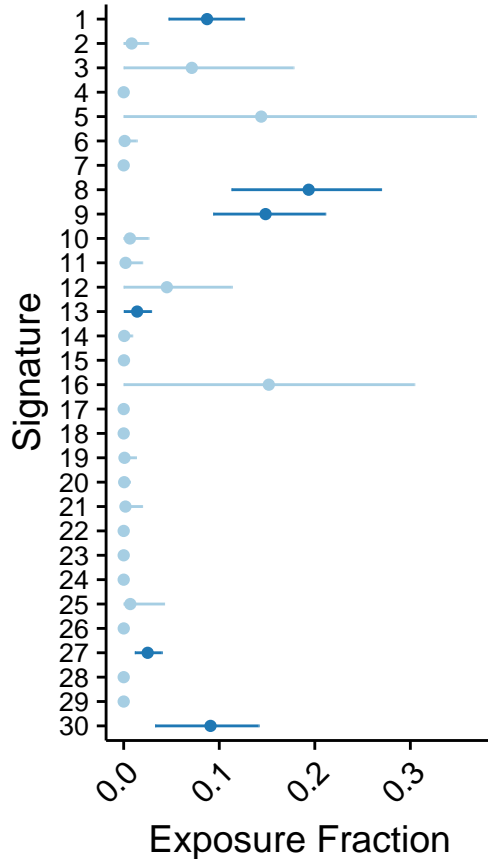

Supplement: Supplementary file 9 — Supplementary Figure S5(PDF 7 kb) [file 41698_2018_50_MOESM9_ESM.pdf]

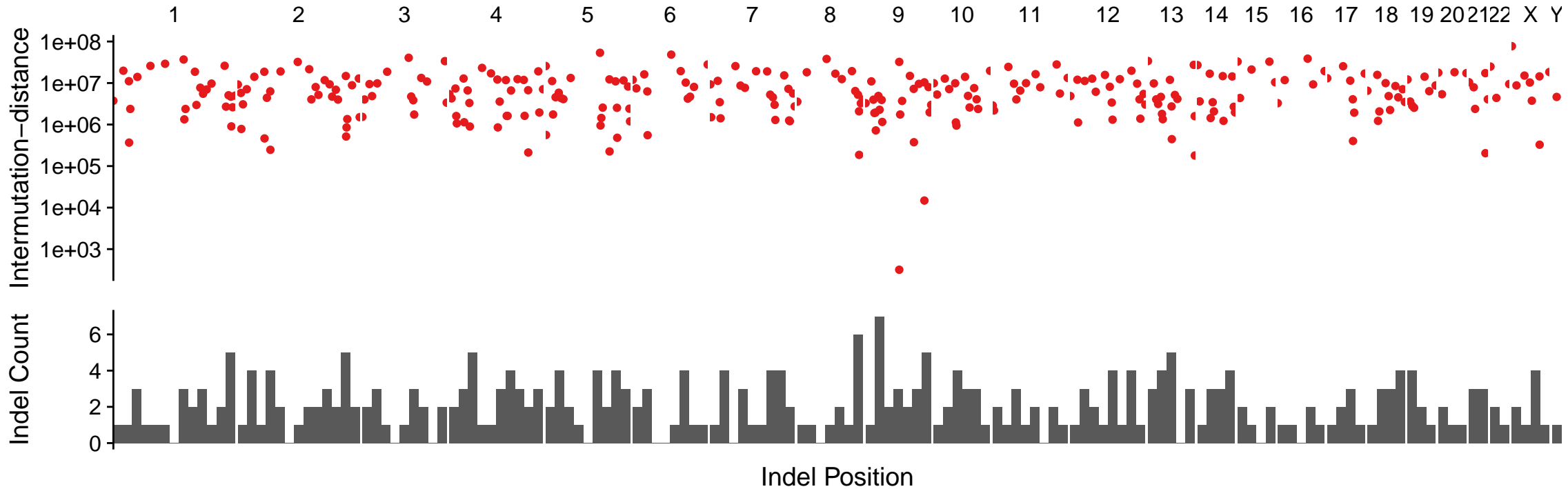

Supplement: Supplementary file 10 — Supplementary Figure S6(PDF 9 kb) [file 41698_2018_50_MOESM10_ESM.pdf]

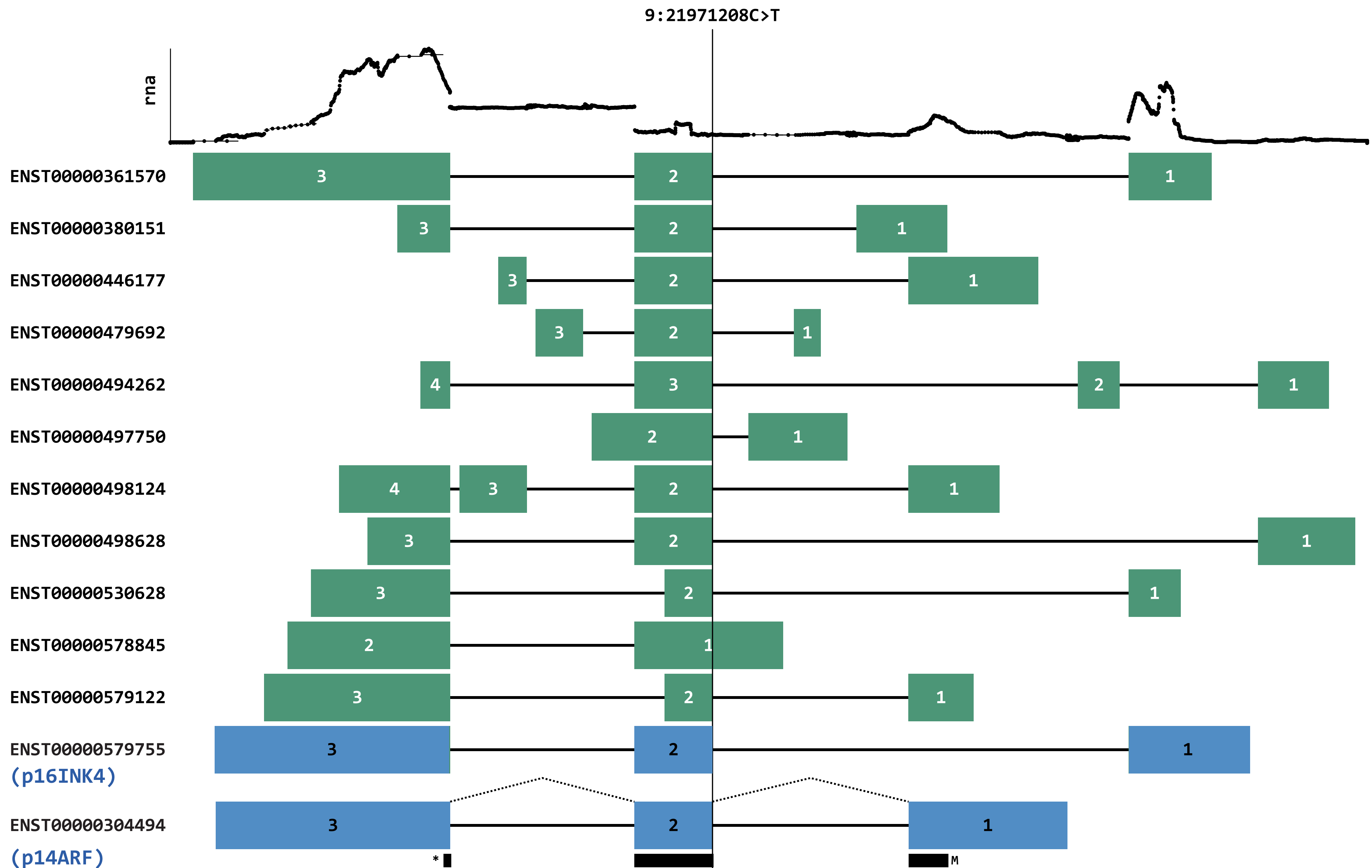

Supplement: Supplementary file 11 — Supplementary Figure S7(PDF 1047 kb) [file 41698_2018_50_MOESM11_ESM.pdf]

# RPKM expression per CDKN2A exon

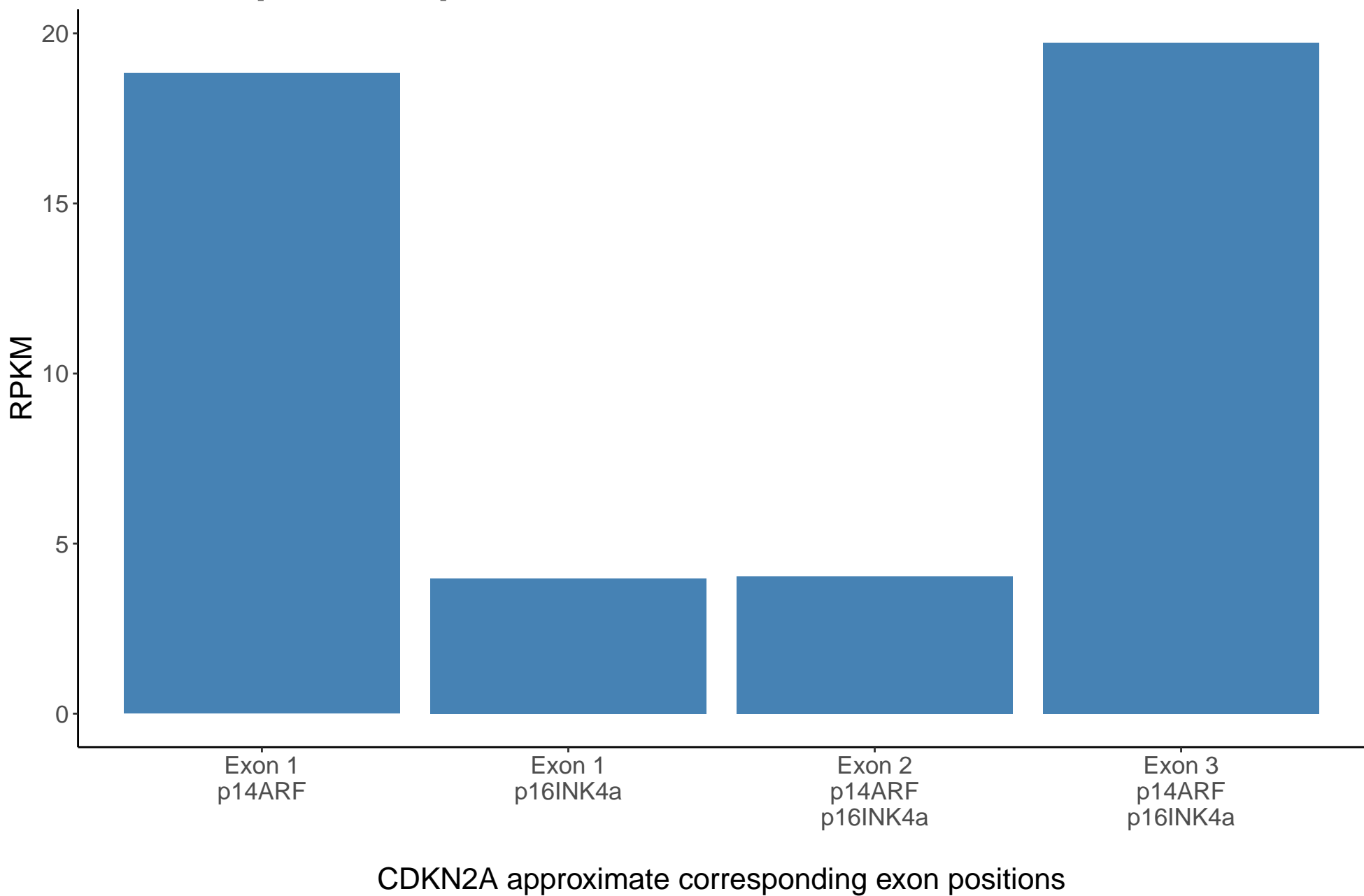

Supplement: Supplementary file 12 — Supplementary Figure S8(PDF 4 kb) [file 41698_2018_50_MOESM12_ESM.pdf]
