# Supplementary material for: Whole genome and whole transcriptome genomic profiling of a metastatic eccrine porocarcinoma
Source: NPJ Precis Oncol. 2018 Mar 19;2:8. doi: 10.1038/s41698-018-0050-5 (PMC5871832; doi:10.1038/s41698-018-0050-5)

Spearman correlation

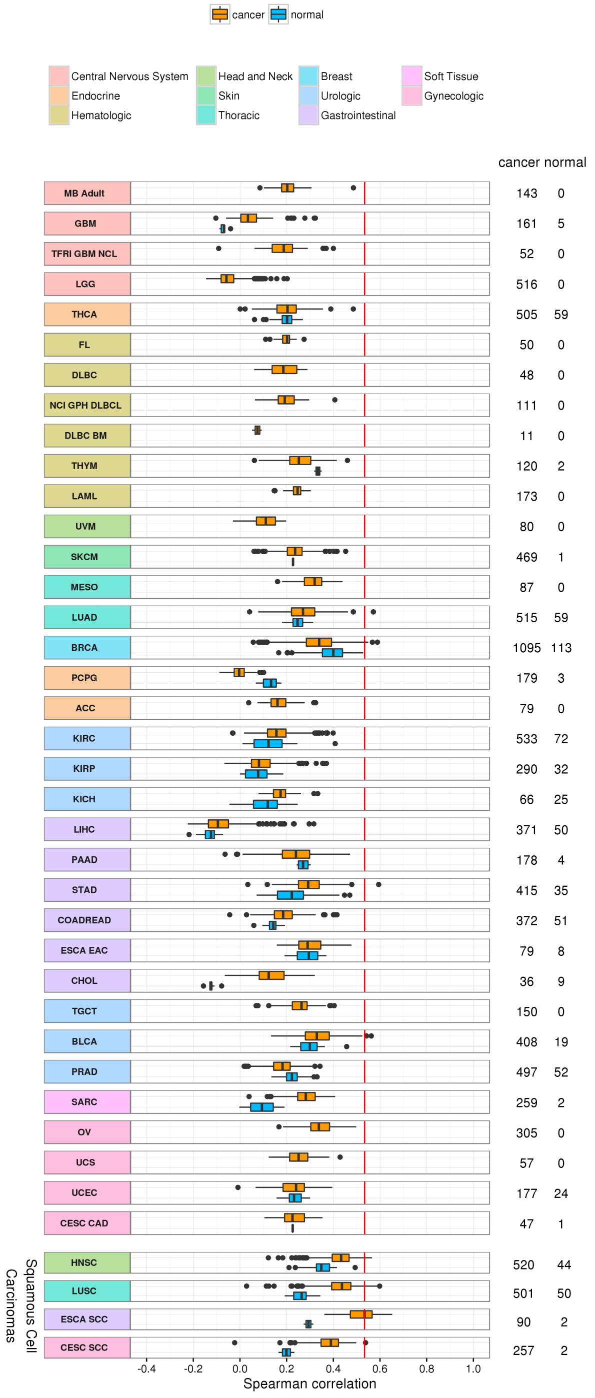

Supplement: Supplementary file 7 — Supplementary Figure S3(PDF 687 kb) [file 41698_2018_50_MOESM7_ESM.pdf]
