# Supplementary material for: Whole genome and whole transcriptome genomic profiling of a metastatic eccrine porocarcinoma
Source: NPJ Precis Oncol. 2018 Mar 19;2:8. doi: 10.1038/s41698-018-0050-5 (PMC5871832; doi:10.1038/s41698-018-0050-5)
Supplement: Supplementary file 13 — Supplementary Figure S9(PDF 129 kb) [file 41698_2018_50_MOESM13_ESM.pdf]

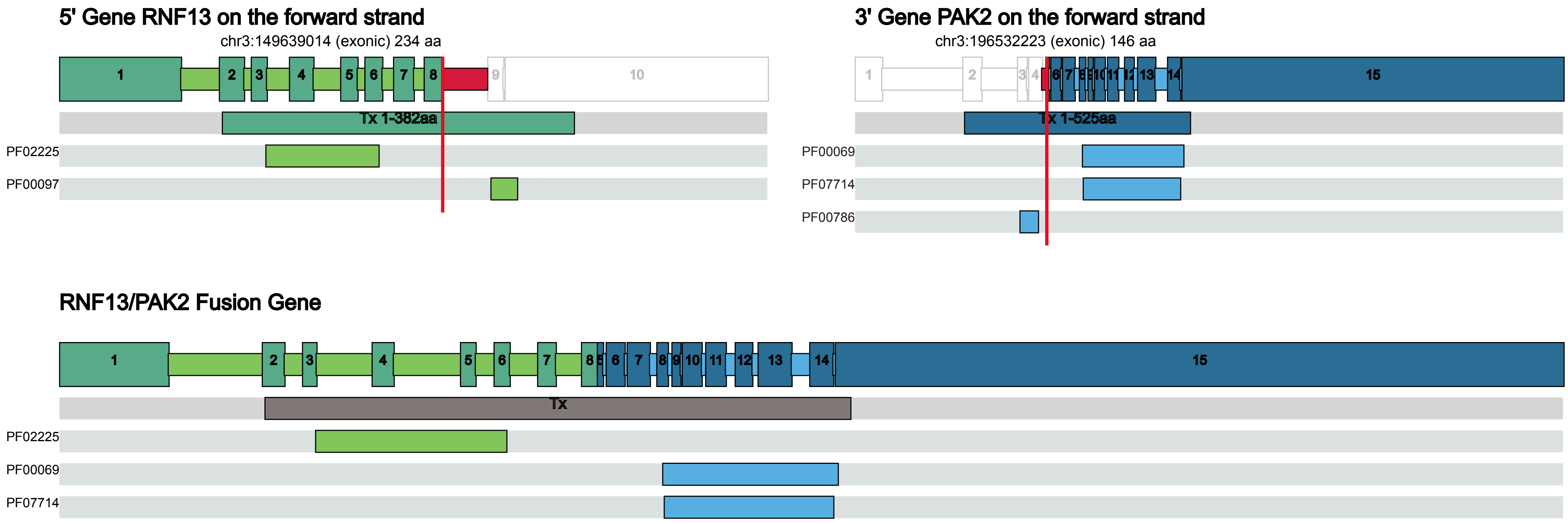
5' Gene RNF13 on the forward strand
chr3:149639014 (exonic) 234 aa
1
2
3
4
5
6
7
8
9
10
Tx 1-382aa
PF02225
PF00097
3' Gene PAK2 on the forward strand
chr3:196532223 (exonic) 146 aa
1
2
3
4
6
7
13
14
15
Tx 1-525aa
PF00069
PF07714
PF00786
RNF13/PAK2 Fusion Gene
1
2
3
4
5
6
7
8
6
7
8
9
10
11
12
13
14
15
Tx
PF02225
PF00069
PF07714
