# Supplementary material for: Whole genome and whole transcriptome genomic profiling of a metastatic eccrine porocarcinoma
Source: NPJ Precis Oncol. 2018 Mar 19;2:8. doi: 10.1038/s41698-018-0050-5 (PMC5871832; doi:10.1038/s41698-018-0050-5)
Supplement: Supplementary file 14 — Supplementary Figure S10(PDF 356 kb) [file 41698_2018_50_MOESM14_ESM.pdf]

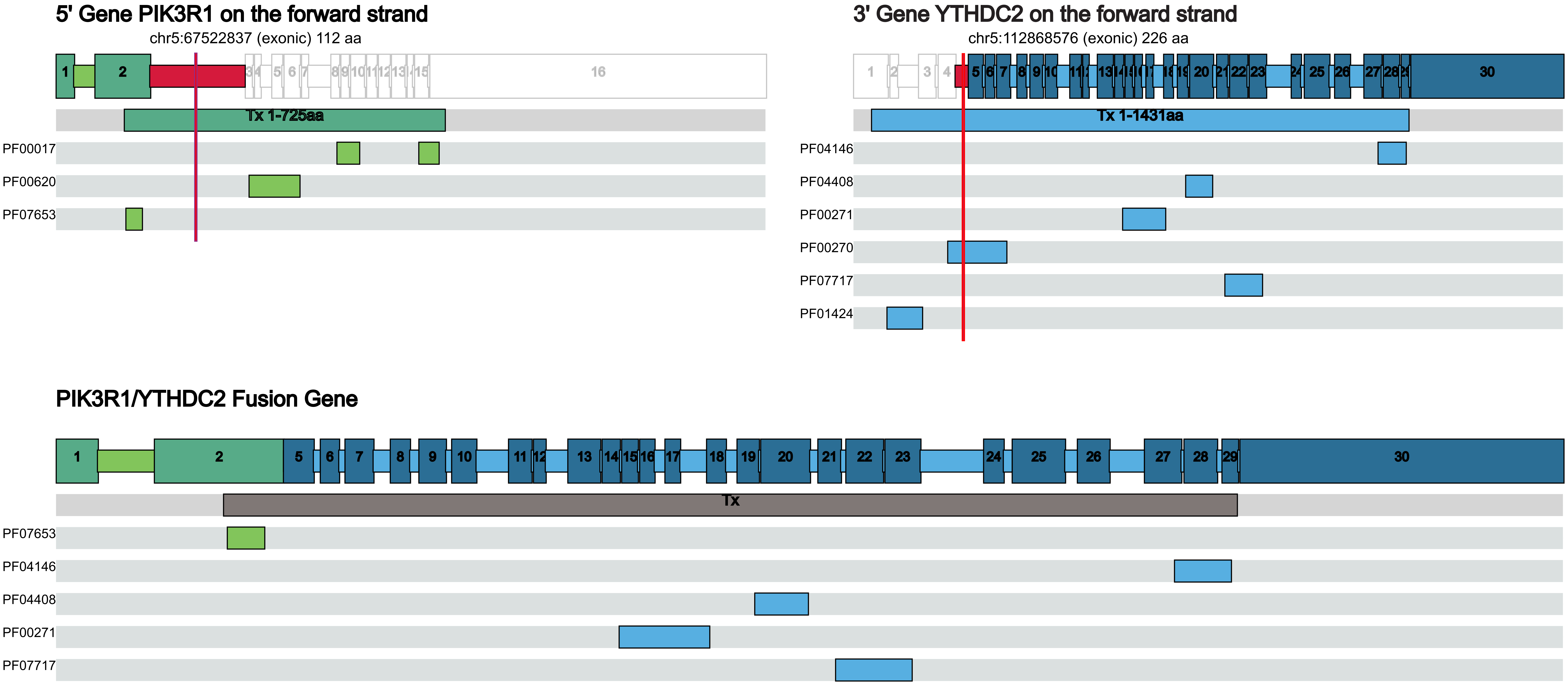

5' Gene PIK3R1 on the forward strand
chr5:67522837 (exonic) 112 aa
Tx 1-725aa
PF00017
PF00620
PF07653
3' Gene YTHDC2 on the forward strand
chr5:112868576 (exonic) 226 aa
Tx 1-1431aa
PF04146
PF04408
PF00271
PF00270
PF07717
PF01424
PIK3R1/YTHDC2 Fusion Gene
Tx
PF07653
PF04146
PF04408
PF00271
PF07717
